# Expression Profiling of Long Noncoding RNA Splice Variants in Human Microvascular Endothelial Cells: Lipopolysaccharide Effects *In Vitro*

**DOI:** 10.1155/2017/3427461

**Published:** 2017-09-25

**Authors:** Imran H. Chowdhury, Hema P. Narra, Abha Sahni, Kamil Khanipov, Casey L. C. Schroeder, Jignesh Patel, Yuriy Fofanov, Sanjeev K. Sahni

**Affiliations:** ^1^Department of Pathology, University of Texas Medical Branch, 301 University Boulevard, Galveston, TX 77555, USA; ^2^Institute for Human Infections and Immunity, University of Texas Medical Branch, Galveston, TX, USA; ^3^Department of Pharmacology, University of Texas Medical Branch, Galveston, TX, USA

## Abstract

Endothelial cell interactions with lipopolysaccharide (LPS) involve both activating and repressing signals resulting in pronounced alterations in their transcriptome and proteome. Noncoding RNAs are now appreciated as posttranscriptional and translational regulators of cellular signaling and responses, but their expression status and roles during endothelial interactions with LPS are not well understood. We report on the expression profile of long noncoding (lnc) RNAs of human microvascular endothelial cells in response to LPS. We have identified a total of 10,781 and 8310 lncRNA transcripts displaying either positive or negative regulation of expression, respectively, at 3 and 24 h posttreatment. A majority of LPS-induced lncRNAs are multiexonic and distributed across the genome as evidenced by their presence on all chromosomes. Present among these are a total of 44 lncRNAs with known regulatory functions, of which 41 multiexonic lncRNAs have multiple splice variants. We have further validated splice variant-specific expression of EGO (NONHSAT087634) and HOTAIRM1 (NONHSAT119666) at 3 h and significant upregulation of lnc-IL7R at 24 h. This study illustrates the genome-wide regulation of endothelial lncRNA splice variants in response to LPS and provides a foundation for further investigations of differentially expressed lncRNA transcripts in endothelial responses to LPS and pathophysiology of sepsis/septic shock.

## 1. Introduction

Uncontrolled systemic inflammation caused by sepsis is one of the leading causes of death and disability throughout the world [1, 2]. Sepsis is defined as a systemic inflammatory response syndrome due to its ability to involve multiple organ systems distant from the site(s) of infection [1, 3]. Exposure of endothelial cells to lipopolysaccharide (LPS) in the cell wall of Gram-negative bacteria plays a central role in the pathophysiology of sepsis [1]. The single, continuous layer of endothelial cells lining the microcirculation constitutes an intricate organ responsible for maintaining an antiadhesive and antithrombotic surface along the blood vessels and regulation of blood flow and vasomotor tone. Interactions between microvascular endothelium and blood-borne endotoxins such as LPS and inflammatory cytokines such as interferon-*γ* result in the shedding of endothelial glycocalyx, increased expression of inflammatory markers (intercellular adhesion molecule-1, vascular cell adhesion molecule-1 (VCAM-1), and interleukin-6 (IL-6)), disruption of gap junctions, and increased vascular permeability [4, 5].

The human genome harbors a large number of sequences coding for RNAs that are not translated but implement regulatory effects on myriad cellular functions. It is rather intriguing that although a majority of the genome is transcribed, a very small fraction of only 2% encodes for the proteins and the rest gives rise to thousands of noncoding RNAs (ncRNAs) lacking protein-coding capacity [6]. In recent years, it has become increasingly evident that ncRNAs are involved in diverse major biological processes, including immune regulation, cell cycle, apoptosis, posttranscriptional and translational regulation, epigenetic modification, and nuclear genome organization [7–11]. ncRNAs are broadly classified into short ncRNAs of <200 nucleotides and long noncoding RNAs (lncRNAs) of lengths varying from >200 nucleotides to tens of kilobases [12]. lncRNAs are highly versatile molecules that can bind to other RNA templates, DNA, and a vast repertoire of proteins, highlighting their regulatory potential in the determination of pathophysiology of various human ailments, including Alzheimer's disease, cardiovascular disease, and cancer [13–16].

The innate immune recognition of bacterial products is orchestrated by a family of transmembrane receptors known as Toll-like receptors (TLRs) [17]. Endothelial cells are known to express a number of TLRs, including TLR4, and display an activated phenotype mediated by a receptor complex consisting of TLR4, cluster of differentiation 14 (CD14), and myeloid differentiation protein-2 (MD-2) in response to LPS [1]. The early, immediate response is governed by the recruitment of myeloid differentiation factor 88 (MyD88), an adaptor protein which initiates a MyD88-dependent pathway culminating in the early activation of nuclear factor-kappaB (NF-*κ*B) and mitogen-activated protein kinase (MAPK) pathways. Parallel activation of a MyD88-independent pathway results in the late-phase activation of NF-*κ*B [18]. Published evidence also documents the ability of LPS to trigger both apoptosis and expression of anti-apoptotic proteins in endothelial cells [19]. Since the activation of endothelial cell signaling and injury by LPS plays a major role in the pathogenesis of bacterial sepsis and septic shock, the molecular mechanisms underlying endothelial responses to LPS have been investigated extensively [20–22]. However, with the exception of a recently published study [22], the regulatory roles of lncRNAs as potential contributors to endothelial cell responses to LPS and determinants of pathophysiological mechanisms of sepsis have not yet been explored in much detail. Moreover, lncRNAs exhibit distinct patterns of expression in different cell types, necessitating the identification and characterization of transcript variants of lncRNAs responsible for the pathogenesis of sepsis syndrome. In an attempt to address this important knowledge gap, we have investigated the cumulative lncRNA signature of microvascular endothelium treated *in vitro* with LPS. Our results not only suggest robust changes in the lncRNA transcriptome of endothelial cells treated with LPS but also reveal differential expression of selective splice variants of lncRNAs with known function(s).

## 2. Materials and Methods

### 2.1. Cell Culture, LPS Treatment, and RNA Isolation

Human dermal microvascular endothelial cells (HMECs) obtained from the Centers for Disease Control and Prevention (Atlanta, GA) were grown in MCDB 131 medium (Caisson's Laboratories) containing 10% fetal bovine serum (Aleken Biologicals), 10 ng/ml epidermal growth factor (Thermo Fisher Scientific), 1 *μ*g/ml hydrocortisone (Sigma), and 10 mM L-glutamine (Thermo Fisher Scientific) [23]. LPS from *Escherichia coli* (*E. coli* O111: B4; Invivogen) was dissolved in sterile water and prepared fresh at the time of use. At approximately 90% confluence in culture, HMECs were treated with LPS (1 *μ*g/ml) for 3 and 24 h. HMECs were used at passage numbers 22 to 25 and routinely tested for mycoplasma contamination at the Tissue Culture Core Facility at the University of Texas Medical Branch (UTMB). RNA-Seq was performed on two independent mock-treated controls and LPS-treated samples (3 and 24 h). Total RNA was isolated by the Tri-Reagent method (Invitrogen) following a combination of the manufacturer's instructions and our previously optimized procedures [24, 25]. Total RNA was treated with DNaseI (NEB) to remove any genomic DNA contamination. The quality of RNA preparations was verified on a bioanalyzer (Agilent Technologies), and samples with an RNA integrity number (RIN) of >9.0 were used for further analysis.

### 2.2. RNA Sequencing

Total RNA was enriched using a Ribo-Zero rRNA Removal kit (Illumina). cDNA libraries were constructed from enriched total RNA, and RNA-Seq was performed on an Illumina HiSeq1500® system at the Molecular Genomics and Sequencing Core facility of the UTMB as 150 base single-end reads. The filtration of resultant reads was performed to ensure inclusion of only high quality sequences in the downstream analysis. The process of filtration included removal of reads containing nucleotide sequences below the quality threshold of 0.05 (using modified the Richard Mott algorithm), unknown nucleotides, and adapters used in the generation of sequencing libraries. The high quality reads thus obtained were trimmed by 15 bases from the 5′ end to reduce nucleotide bias from their origin. All high-quality reads retained after trimming were mapped to *Homo sapiens* RefSeq coding genes (GRch37/hg19) using CLC Genomics Workbench 9.0.1 (http://www.clcbio.com), and the reads mapping to known protein-coding genes (PCGs) were excluded from further analysis. Finally, the remaining reads, which did not map to the protein-coding transcriptome, were mapped to the human ncRNA database (NONCODE_V4; www.noncode.org) [26] to identify differentially expressed ncRNAs in reponse to LPS treatment. Mapping to both RefSeq genes and ncRNAs was performed allowing up to 9 base mismatches (94.4% identity) as determined by global alignment scoring. Samples were grouped according to their designation as either control (mock treated) or treated (LPS treatment). The RNA-Seq data was further analyzed and normalized by calculating reads per kilobase million (RKPM) for each transcript using the formula below [27].

(1) $$\begin{array}{c} \frac{Total\;exon\;reads}{Mapped\;reads\;\left(millions\right)\times exon\;length\;\left(KB\right)\;}. \end{array}$$

Finally, fold changes for PCGs and ncRNA transcripts (excluding short ncRNAs of <200 bp) at 3 and 24 h posttreatment were determined using mock-treated controls as the baseline.

### 2.3. Cataloging of lncRNAs

Differentially expressed lncRNA transcripts in our RNA-Seq data were catalogued based on their chromosomal and strand origin according to NONCODE database (NONCODE_V4) and grouped based on their chromosomal location (Chr 1–22, Chr 23 (X and Y), and mitochondrial DNA) and origin of the strand (sense or antisense). All lncRNAs originating from X and Y alleles of chromosome 23 were merged together and named as XY. The length of differentially expressed lncRNA transcripts and their exon numbers were also captured from the NONCODE database. We grouped lncRNA transcripts based on their length into four categories of 200–500 bp, 501–2000 bp, 2001–5000 bp, and above 5000 bp. Similarly, differentially expressed lncRNA transcripts were categorized as uniexonic, biexonic, and multiexonic based on the exon numbers. Sequences of known lncRNAs with assigned function(s) were downloaded from lncRNAdb (http://www.lncrnadb.org) [28] and blasted against the sequences for ncRNAs in the NONCODE database. We thus captured NONCODE IDs for known lncRNAs and also recorded all of their transcript variants from the NONCODE database. To further understand the expression profile of PCGs overlapping with the differentially expressed lncRNAs identified in this study, we segregated the lncRNA transcripts based on their genomic location. The expression of PCGs from the exonic or antisense lncRNA transcripts was determined based on RPKM values as described above. Finally, to compare our results to the expression profile of lncRNAs in HUVECs (human umbilical vein endothelial cells) during LPS treatment [22], we downloaded the sequences for differentially expressed lncRNAs in this study from LNCipedia (LNCipedia.org) and NCBI and performed BLAST analysis of these lncRNAs in the NONCODE database to obtain their corresponding NONCODE IDs.

### 2.4. Quantitative Real-Time PCR (qRT-PCR)

**Figure 1:**
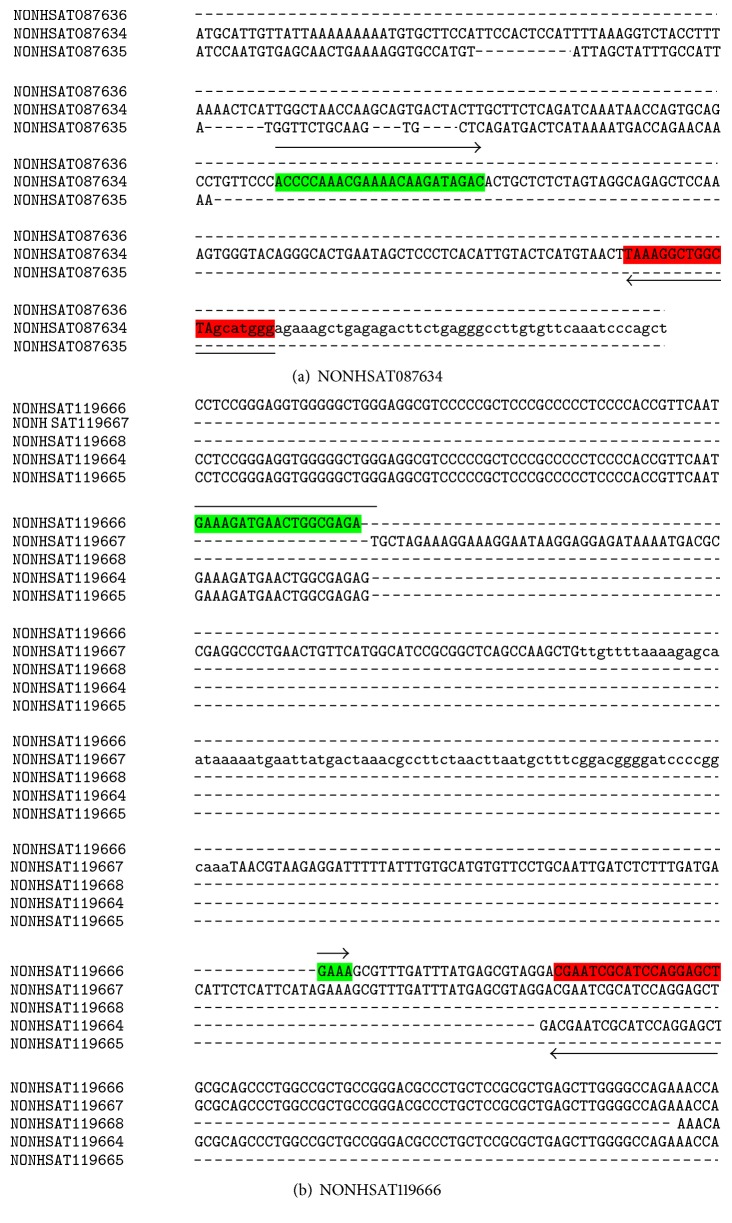
Schematic of (a) NONHSAT087634 and (b) NONHSAT119666 splice variant specific primer selection.

SYBR green-based quantitative polymerase chain reaction (qRT-PCR) assays were carried out for further validation of selected lncRNA transcripts from the RNA-Seq analysis on a StepOnePlus Real-time PCR System (Applied Biosystems). Briefly, DNaseI-treated RNA (1 *μ*g) was reverse transcribed using a cDNA synthesis kit (Applied Biosystems) and qPCR was performed using splice variant-specific primers designed using Primer Express 3.0.1 software (Applied Biosystems). The sequences of splice variant-specific primers and the schematics employed for their selection are presented in Table 1 and Figure 1. Full-length sequence alignments of lncRNA transcript variants are presented in the Supplementary File. The PCR reactions were performed in triplicate, and the expression of transcripts normalized against the housekeeping gene 18S rRNA. The level of expression and relative quantification were determined via calculations based on the 2^−∆Ct^ [2^(−ΔCt of treatment)^/2^(−ΔCt of control)^] method.

### 2.5. RefSeq Pathway Analysis

To identify differentially regulated protein-coding gene pathways in HMECs during LPS treatment, we mapped our RNA-Seq reads to the human RefSeq database (GRch37/hg19) following same parameters as described above for ncRNAs. PCGs showing ≥3-fold up- or downregulation were further subjected to Ingenuity® Systems Pathway Analysis (IPA; Ingenuity Systems, Redwood City, CA, USA; http://www.ingenuity.com) to identify significantly regulated canonical pathways in endothelial cells during LPS treatment in direct comparison to the mock-treated controls. Only pathways exhibiting significant changes (*P* ≤ 0.05) were considered for the analysis.

### 2.6. Statistical Analysis

The D'Agostino and Pearson omnibus normality test was performed to test the normal distribution of data. Comparisons between the unmatched groups were done by the Mann–Whitney *U* test. GraphPad Prism version 5 (GraphPad Software Inc., San Diego, California) was used for the statistical analysis. Statistical significance was accepted at the *P* value of ≤0.05.

## 3. Results

**Figure 2:**
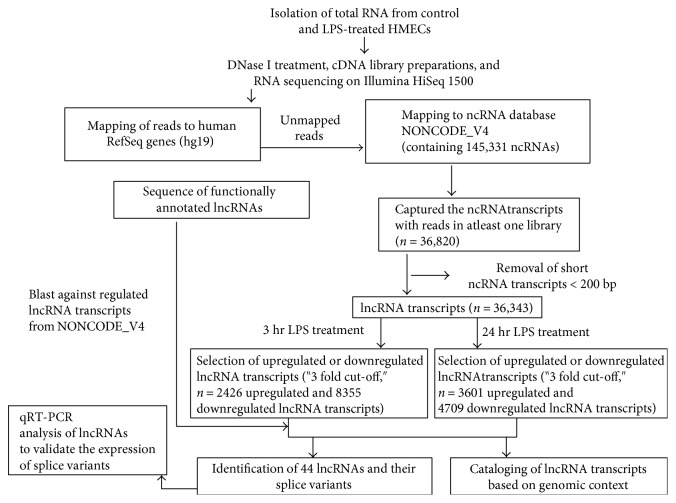
Schematic illustration of the procedure for identification, cataloging, and validation of lncRNA transcripts.

To catalogue alterations in the lncRNA expression profile of HMECs subjected to LPS treatment for 3 h and 24 h, we first performed RNA sequencing. Sequencing produced approximately 28–37 million reads per library, of which nearly 31–36% and 11–15% of the reads mapped to mRNAs and ncRNAs, respectively (Supplementary Table 1 available online at https://doi.org/10.1155/2017/3427461). Of the total RNA in eukaryotic cells, mRNAs and rRNAs represent approximately 5 and 80%, respectively, whereas the remaining 15% includes other RNA molecules comprised of spliceosomal RNAs, snoRNAs, nonpolyadenylated RNAs, tRNAs, mitochondrial RNAs, and other specialized RNAs such as LINE/SINE or B-element RNAs which are abundantly expressed and mapped to the repeat regions of the genome [29]. Similar to the results observed in our RNA-Seq datasets, a recent study documents the mapping of about 30–60% of the reads to rRNAs despite the application of enrichment procedures [30]. In the present study, we consistently achieved enrichment of mRNA and lncRNA reads following Ribo-Zero rRNA removal and any remaining reads mapping to rRNAs and tRNAs were not included in a downstream analysis. The total reads thus obtained (sans those originating from rRNAs and tRNAs) from untreated controls and LPS-treated HMECs were first mapped to the RefSeq genes of human genome (GRch37/hg19) to subtract the reads mapping to the protein-coding transcriptome. The remaining reads were then mapped to a total of 145,331 ncRNA transcripts annotated in the NONCODE database. Among these, we selected only the ncRNA transcripts clearly represented by a noticeable number of reads in at least one of our sample categories, that is, untreated and LPS-treated HMECs at 3 h and 24 h, allowing us to narrow down the number of ncRNA transcripts to 36,820. At this point, we only retained ncRNA transcripts with a length of ≥200 bp, leaving a total of 36,343 lncRNA transcripts in our dataset. The schematics employed for the deduction of lncRNA repertoire are presented in Figure 2. Further analysis of changes in the expression of lncRNAs using their basal levels in untreated HMECs (controls) allowed us to identify 2426 and 8355 lncRNAs that were either up- or downregulated in LPS-treated HMECs at 3 h. Through an identical analysis, a total of 3601 and 4709 lncRNAs were found to be either up- or downregulated at 24 h.

**Figure 3:**
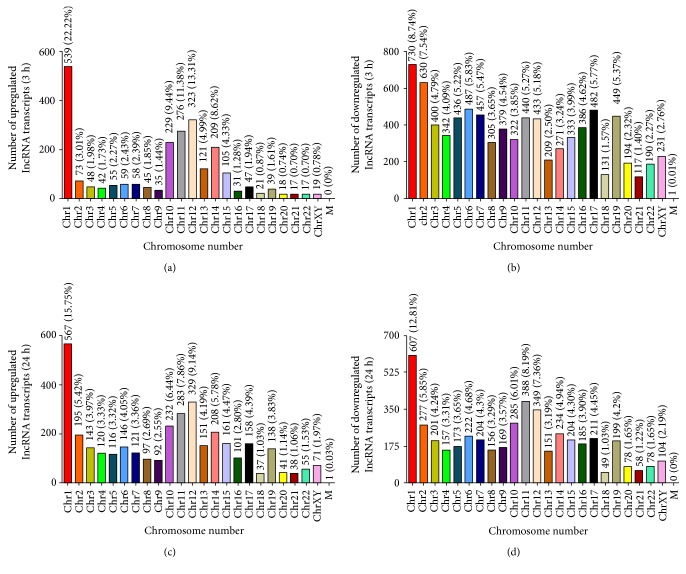
Chromosome-wise distribution of differentially expressed lncRNA transcripts in HMECs after LPS treatment. (a) Upregulated transcripts after 3 h. (b) Downregulated transcripts after 3 h. (c) Upregulated transcripts after 24 h. (d) Downregulated transcripts after 24 h.

**Figure 4:**
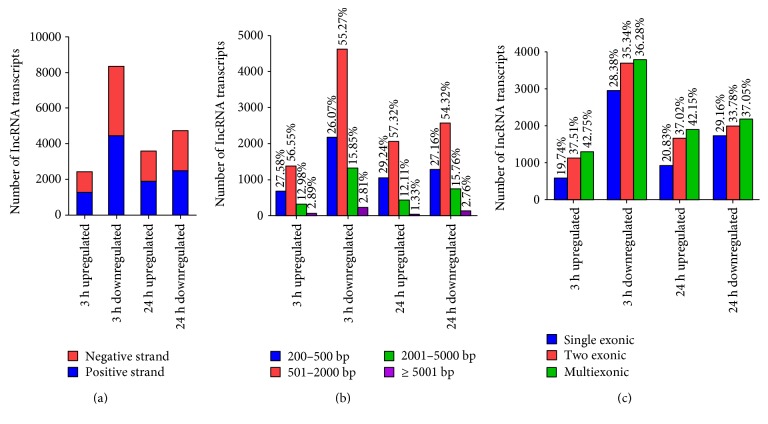
Cataloging of differentially expressed lncRNA transcripts from LPS-treated HMECs based on (a) strand-specific origin, (b) lengthwise distribution, and (c) number of exons.

Next, we focused our attention on differentially expressed lncRNAs (≥3 fold) at 3 and 24 h following LPS treatment by determining their chromosomal location (Figure 3). Long noncoding RNAs modulated upwards and downwards due to LPS treatment were abundant and distributed throughout all the chromosomes. Also, a majority of upregulated lncRNAs at 3 and 24 h posttreatment were located on chromosomes 1, 10, 11, and 12 (Figures 3(a) and 3(c)). On the other hand, lncRNAs displaying downmodulation at 3 h were more or less evenly distributed across all chromosomes (Figure 3(b)) but originated predominantly from chromosomes 1, 10, 11, and 12 at 24 h (Figure 3(d)).  Figure 4 displays the distribution of lncRNAs in our test samples based on the strand of their origin, overall length, and exonic composition. At a glance, subsets of lncRNA-coding sequences undergoing changes in their expression following LPS treatment were almost evenly distributed on the positive (leading) and negative (lagging) strands of the genome (Figure 4(a)). Our results further suggest that nearly 58% of both up- and downregulated lncRNA transcripts range from 501–2000 bp, while there are relatively few transcripts of >5001 bp (Figure 4(b)). In regard to their genomic composition, about 25 to 30% differentially expressed lncRNA transcripts are uniexonic, while the remaining have two or more exons (Figure 4(c)).

**Figure 5:**
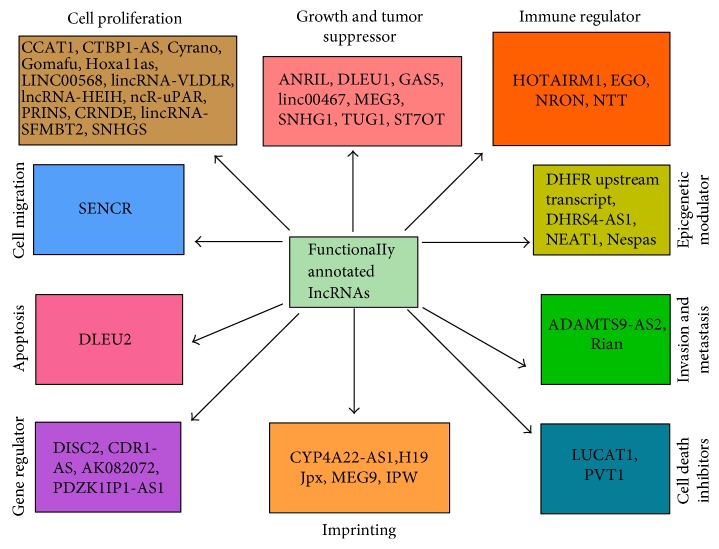
Identification of functionally annotated lncRNAs in HMECs treated with LPS for 3 and 24 h. Differentially expressed lncRNAs belong to different categories based on publicly available databases and published reports.

**Figure 6:**
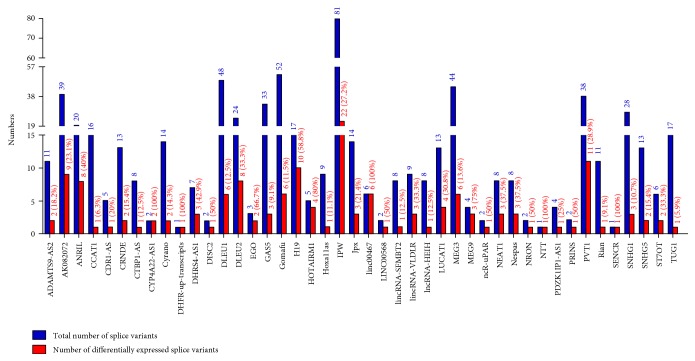
Bar diagram represents total splice variants available for 44 functionally annotated lncRNAs and their respective differentially expressed splice variants in HMECs after LPS treatment.

Since very little is known about the functions of lncRNAs in the endothelium and our analysis revealed extensive modulation of their expression, we next focused our efforts on identifying those with a defined functional role in the determination of endothelial responses *in vitro* or *in vivo*. To accomplish this, sequences of functionally annotated lncRNAs were acquired from lncRNAdb [28] and blasted against the NONCODE database. Subsequent search of such lncRNAs in our datasets enabled us to capture a total of 44 lncRNAs that were either up- or downregulated in LPS-treated HMECs at 3 h or 24 h. The characteristic features of these lncRNAs, including their chromosomal location, the number of splice variants, and expression pattern, are detailed in Table 2 and Supplementary Table 2. Functionally, these lncRNAs are involved in the regulation of cell proliferation, growth, and migration; determination of immune responses, modulation of apoptosis, invasion, and metastasis; control of epigenetic modifications; and genomic imprinting (Figure 5). Intriguingly, a total of 41 lncRNAs (out of 44 identified through systematic analysis) have multiple splice variants and are multiexonic (Table 2 and Supplementary Table 2). We also observed differential expression of splice variants belonging to the same lncRNA, suggesting selective modulation of the transcript variants after LPS treatment (Supplementary Table 2).  Figure 6 shows the total number of splice variants available for 44 functionally annotated lncRNAs and the proportion of differentially expressed splice variants in HMECs after LPS treatment at 3 and 24 h. A comparative analysis of our dataset with that of LPS-treated HUVECs in a recently published study [22] enabled the identification of 15 lncRNAs expressed in response to LPS treatment (Table 3). Among these, only one (NONHSAT140176) out of ten transcript variants of lncRNA AL132709.5 displayed higher expression at 24 h. Similarly, only one of seven splice variants of lncRNA AC068282.3, namely, NONHSAT074153, also had higher expression at 24 h in comparison to its basal level in untreated controls. In contrast, both splice variants for CLDN10-AS1 (NONHSAT034761 and NONHSAT034762) were upregulated at 24 h in LPS-treated HMECs. Splice variants of CTC-459I6.1, RP11-138B4.1, and RP11-676J12.6 were not detectable in our datasets at both 3 and 24 h post-LPS treatment. Interestingly, three of eight splice variants of BX284650.1 had no detectable expression, yet the remaining 5 transcript variants showed a variable (either up- or downregulation) pattern of expression in response to LPS. The expression of lncRNA XLOC_006311 (NONHSAT124447) exhibited downregulation at 24 h after LPS treatment. Thus, consistent with our findings, a similar analysis of the expression profile of lncRNAs differentially expressed in human umbilical vein endothelial cells (HUVECs) exposed to LPS also suggests that all splice variants belonging to the same lncRNA are not uniformly expressed [22].

Of the 44 lncRNA candidates identified in this study, 22 are from genomic loci of PCGs (18 antisense, 1 antisense/exonic, 2 exonic, and 1 sense/not exonic), while the remaining 22 originate from the intergenic regions. The expression of PCGs overlapping with these lncRNAs was either downregulated or remained unchanged. Specifically, the expression of ADAMTS9 (ADAM metallopeptidase with thrombospondin type 1 motif 9), HOXA11a (homeobox A11a), and PDE7B (phosphodiesterase 7B) harboring lncRNAs ADAMTS9-AS2, Hoxa11as, and NTT on the respective antisense strand was compromised, while that of ITPR1 on the opposite strand of lncRNA EGO was unaltered at both 3 or 24 h post-LPS treatment (Supplementary Table 3).

**Figure 7:**
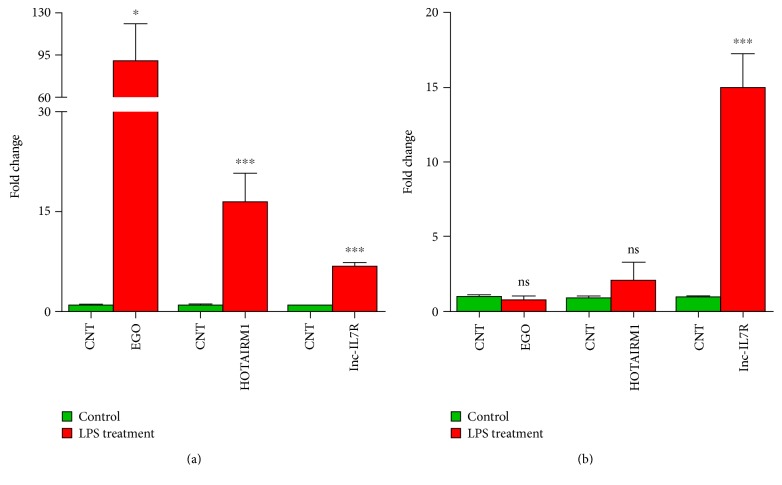
Quantitative RT-PCR for EGO (NONHSAT087634), HOTAIRM1 (NONHSAT119666), and lnc-IL7R expression in HMECs following LPS treatment for (a) 3 h and (b) 24 h (^∗^*P* ≤ 0.05 and ^∗∗∗^*P* ≤ 0.001; and ns = nonsignificant).

EGO, HOTAIRM1, and lnc-IL7R are three lncRNAs with known regulatory functions that were found to be differentially expressed in endothelial cells after LPS treatment. EGO and HOTAIRM1 have 3 and 5 splice variants, respectively. Among them, 2 splice variants of EGO and 4 splice variants of HOTAIRM1 were differentially expressed in response to LPS treatment for 3 and 24 h. To further validate splice variant-specific expression of EGO (transcript NONHSAT087634) and HOTAIRM1 (transcript NONHSAT119666) lncRNAs, we performed qRT-PCR analysis. In agreement with the findings from our RNA-Seq data, the expression of both NONHSAT087634 and NONHSAT119666 transcripts was significantly upregulated at 3 h post-LPS treatment. At 24 h, the expression levels of both NONHSAT087634 and NONHSAT119666 transcripts declined and were not significantly different from the basal level in mock-treated HMECs (Figures 7(a) and 7(b)). Additionally, lnc-IL7R (a known lncRNA involved in the TLR-2/4 signaling pathway) was significantly upregulated at the both 3 and 24 h post-LPS treatment (Figure 7).

Finally, Ingenuity Pathway Analysis revealed a number of endothelial cell signaling pathways activated in response to LPS treatment for 3 and 24 h (Tables 4 and 5). As expected, the pathways involved in the determination of granulocyte/agranulocyte adhesion, death receptor signaling, IL-17 signaling, TNFR1/2 signaling, communication between innate and adaptive immune cells, signaling pathway underlying the cytokine-mediated crosstalk among different types of immune cells, role of cytokines in mediating communication between immune cells, IL-6 signaling, inflammasome pathway, role of pattern recognition receptors in recognition of bacteria and viruses, and apoptosis signaling pathways were activated in cells treated for 3 h. On the other hand, granulocyte/agranulocyte adhesion, role of cytokines in mediating communication between immune cells, high-mobility group box 1 protein (HMGB1) signaling, IL-17 signaling, role of pattern recognition receptors in recognition of bacteria and viruses, MAPK signaling, IL-6, apoptosis, chemokine signaling, and atherosclerosis signaling pathways were activated at 24 h. Molecules involved in these important signaling pathways contributing to the downstream cellular responses are presented in Supplementary Tables 4 and 5. Importantly, the pathways determining the role of macrophages, fibroblasts and endothelial cells in rheumatoid arthritis, atherosclerosis signaling, glucocorticoid receptor signaling, HMGB1 signaling, hepatic fibrosis/hepatic stellate cell activation, and the expression of inflammatory cytokines CXCL8, IL-6, and adhesion molecule VCAM-1 were significantly higher during LPS treatment at both 3 and 24 h.

## 4. Discussion

The strategic location of vascular endothelium at the interface of circulating blood and underlying tissues also renders it highly susceptible to injury or infection [1]. In addition to regulating blood flow through the maintenance of an active antithrombotic surface, an important function of the endothelial cells is to provide a semipermeable barrier that facilitates the transit of the plasma and cellular constituents throughout the vasculature. LPS is a characteristic component of the outer membrane of Gram-negative bacterial cell wall, a pathogen-associated molecular pattern allowing the host cells to recognize bacterial invasion, and a prototypical trigger of sepsis due to the biological response stimulating or modifying activities of lipid A [31, 32]. Also, LPS directly affects the endothelial barrier function of the blood vessels and induces potent inflammatory responses broadly characterized as a “cytokine storm” [33]. Such an enormous production of inflammatory mediators from endothelial cells after activation by LPS contributes to the pathophysiology of sepsis and endotoxic shock. It is well established that LPS activates cellular signaling mechanisms through interactions with surface TLR4, CD14, and MD2 [34] and that LPS from some bacteria, for example, *Leptospira interrogans*, can also signal through the TLR2 pathway [35].

It is now well appreciated that despite the transcription of a major proportion (>80%) of human genome, PCGs account for only ~2%, while an overwhelming majority is transcribed into ncRNAs lacking protein-coding capacity [36]. Of these, ncRNAs longer than 200 nucleotides and endowed with the ability to interact with mRNAs, DNA, and a broad array of proteins are defined as lncRNAs [37, 38]. Thus, in contrast to microRNAs, lncRNAs bear the potential to regulate gene expression at the levels of transcription, posttranscription, and epigenetics [7–9]. Importantly, the expression of lncRNAs occurs in a cell-, tissue-, and species-specific manner [39–41]. Although the roles of lncRNAs in the pathophysiology of cardiovascular diseases and cancer have garnered significant attention, the potential for their involvement in endothelial responses is only beginning to be elucidated. A recent study based on microarray profiling suggests differential expression of a number of lncRNAs and provides a snapshot of lncRNA/mRNA transcriptome of macrovascular human umbilical vein endothelial cells (HUVECs) during LPS treatment [22]. In the present study, we conducted an RNA-Seq-based investigation of the expression profile of LPS-responsive lncRNAs in human microvascular endothelial cells to identify a total of 10,781 and 8310 lncRNA transcripts that were either positively or negatively regulated at 3 and 24 h post-LPS treatment, respectively. This pool of induced or suppressed lncRNAs was then subjected to further cataloging on the basis of their strand of origin, chromosome-wise origin and distribution pattern, and exon composition. Interestingly, a majority of lncRNAs undergoing changes in their expression levels were determined to be multiexonic. Multiexonic lncRNAs composed of varying combination of exons can be differentially expressed in a cell/tissue-specific manner, and accumulating evidence suggests tissue-specific expression of different splice variants of individual lncRNAs [42, 43]. Accordingly, the findings of this study further demonstrate differential expression of only selective splice variants of LPS-responsive lncRNAs in microvascular endothelium.

A “search and identify” strategy based on the comparative analysis of our datasets with lncRNAdb, a database listing functionally annotated lncRNAs [28], allowed us to identify 44 lncRNAs modulated as a consequence of LPS treatment. Among these, a subset of regulatory lncRNAs, namely, HOTAIRM1, EGO, NRON and NTT, is involved in immune regulation and the progression of disease [44–47], whereas others such as NEAT1, Nespas, DHFR upstream transcript, and DHRS4-AS1 are known as epigenetic regulators [48–51]. In addition, H19, Jpx, and MEG9 have been implicated in the regulation of genomic imprinting [52–54]. We were able to further identify other lncRNAs, namely, Gomafu, lncRNA-HEIH, Hoxa11as, and PRINS, that reportedly function as inducers of cell proliferation [55–58]. In contrast, lncRNAs, such as ANRIL, GAS5, MEG3, and TUG1, have been shown to be involved in growth suppression [59–62]. It is important to also consider, however, that a recent study documents the involvement of TUG1 in promoting cell growth and chemoresistance in small cell lung cancer [63]. Together, these findings suggest the possibility of context-dependent multifunctional roles of lncRNAs. Intriguingly, a majority of regulated lncRNAs are also expressed as multiple splice variants and only selective splice variants of a particular lncRNA display differential expression in our experimental model system (Table 2 and Supplementary Table 2). It is well known that splicing constitutes a critically important step in the processing of mature RNAs within eukaryotic cells. Through this phenomenon, introns present in the premature mRNA are removed, yielding organization of exons in different/specific combinations and diversity of encoded protein sequences. Spliceosome and splicing factors are, therefore, of critical importance in determining the splice variants of transcriptome. Like premature mRNA, lncRNAs are also processed in different exonic combinations after splicing of introns [64–66] and expression of splice variants of lncRNAs for 16 tissue types has been documented in human body map 2 [26, 67]. Moreover, an array of biological functions for the splice variants of lncRNAs such as MALAT1, ANRIL, Nespas, SOX2OT, and GAS5 is also being realized and reported in the literature [67–71]. Our analysis demonstrates differential expression of 6 splice variants of MEG3 amongst a total of 44 for this lncRNA (Table 2 and Supplementary Table 2). Similarly, 2 out of 3 possible splice variant transcripts of EGO and 4 of 5 splice variants of HOTAIRM1 display regulation at either 3 or 24 h following LPS treatment, indicating differential modulation of selective transcripts. Importantly, comparative bioinformatics analysis reveals that our data are in agreement to a large extent with the lncRNA profile of LPS-treated HUVECs, since we identified differential expression of selective transcript variants for a majority (15 of 20) of lncRNAs determined to be either highly up- or downregulated in that study [22]. A critical gap in our knowledge that needs to be addressed is that functional implications for a majority of these lncRNAs in the biology of vascular endothelium remain unknown. Also, in light of the concept of endothelial heterogeneity, the possibility of varying expression in different types of tissue- and vessel-specific endothelial beds and variations in downstream biological effects needs to be investigated in further details.

A number of previous studies have highlighted the importance of HOTAIRM1 and EGO in immune cell activation. *HOTAIRM1* (*HOX antisense intergenic RNA myeloid 1*), a lincRNA located in the *HOXA* genomic cluster, modulates the expression of genes involved in myeloid differentiation. Recent evidence suggests increased expression of HOTAIRM1 in cardiomyocytes treated *in vitro* with LPS or those isolated from mice receiving LPS to induce sepsis [72]. EGO, another lncRNA highly expressed in the bone marrow, is involved in eosinophil differentiation of CD34^+^ hematopoietic progenitor cells through regulation of expression of eosinophil granule protein, although its mode of action has not been defined yet [45]. Considering the contributions of these lncRNAs to the development and activation of innate immune responses, we further ascertained altered expression of the most highly regulated splice variant NONHSAT087634 of EGO and NONHSAT119666 of HOTAIRM1 by qRT-PCR to consolidate our findings from RNA-Seq. Also, published evidence implicates an important role for MALAT1 in enhancing the expression of TNF-*α* in cardiomyocytes after LPS treatment [73] and suggests that lincRNA-Tnfaip3 aids transcription factor NF-*κ*B in the induced expression of inflammatory genes in mouse macrophages [74]. LPS treatment also induces the expression of lncRNA NEAT1, which has been shown to be activated in viral infections and is responsible for transcription of inflammatory cytokines through the TLR4 signaling pathway [75, 76]. Although none of the transcript variants of MALAT-1 or lincRNA-Tnfaip3 are activated in LPS-treated HMECs under experimental conditions used in our study, we found that 1 out of 8 splice variants of NEAT-1 shows upregulation at 3 h posttreatment. Interestingly, NEAT1 has been associated with antiviral gene transcription and induction of cytokines such as IL-8. Our findings on the expression of lnc-IL7R, another lncRNA relatively abundant in inflammatory cells, are in agreement with a published report of its expression in HUVECs and THP1 cells in response to LPS and evidence demonstrating that targeted expression and/or activation of lnc-IL7R in LPS-treated cells diminishes the inflammatory response as reflected by decreased expression of proinflammatory cytokines IL-6 and IL-8 and cell adhesion molecules E-selectin and VCAM-1 [77]. Thus, given the stimulus and cell type specificity of lncRNAs, our findings in conjunction with the published evidence suggest potentially important roles for HOTAIRM1, EGO, NEAT1, and lnc-IL7R in endothelial cell responses to LPS.

Endothelial activation, injury, and dysfunction represent major contributory mechanisms underlying the pathogenesis of endotoxic shock and sepsis. Hence, the expression profiles of PCGs in vascular endothelial cells activated by LPS have been extensively investigated [20–22]. In an attempt to decipher the possibility of a correlation between the expression of lncRNAs and their adjoining PCGs, we analyzed our datasets to determine the expression of lncRNA transcripts from the loci of TLR2, TLR4, MD2, and CD14 PCGs. Intriguingly, while none of the lncRNA transcripts originating from the TLR2 and TLR4 genomic loci displayed altered expression, there were no annotated lncRNA transcripts in the genomic loci for CD14 and MD2. Exonic and antisense lncRNAs have been reported as positive and negative regulators of PCGs by diverse mechanisms such as imprinting, epigenetic regulation, splicing, nuclear/cytoplasmic trafficking, and translation [38]. Of the 44 functionally annotated lncRNAs identified in this study, 22 originate from either the sense or antisense strand for the PCGs. Keeping in mind that overlapping lncRNAs might also play a role in regulation of PCGs from the coding or opposite strand, we further examined the expression profile of these genes in our RNA-Seq dataset. A positive correlation between ADAMTS9-AS2 lncRNA and its antisense PCG ADAMTS9, responsible for cell migration, has recently been demonstrated [78]. Accordingly, we noticed that the expression of both transcripts of ADAMTS9-AS2 lncRNA changes with the corresponding mRNA at 3 and 24 h. We further observed a similar expression pattern for the genes HOXA11a and PDE7B antisense to Hoxa11as and NTT lncRNAs, respectively. Wagner et al. have recently dissected the functional importance of EGO in eosinophils [45]. Although genomic loci of EGO are nested within the intronic region of the ITPR1 gene on the opposite strand, the expression of this lncRNA does not correlate with the expression of mRNA. Interestingly, the level of ITPR1 mRNA does not change after LPS treatment in our dataset, supporting a previous finding [45]. Ingenuity Pathway Analysis revealed that after LPS treatment, a number of differentially regulated pathways related to immune response, apoptosis, proliferation, and cell adhesion were activated at 3 and 24 h, and our findings (Figure 5()) indicate that a number of functionally annotated lncRNAs might be associated with these pathways.

The limitation of this study is that a majority of the lncRNA splice variants have not been characterized in detail as of yet, thus limiting our efforts to functionally annotate their role in vital cellular functions such as immune regulation, epigenetic modulation, cell proliferation, and growth suppression. Therefore, further investigations aimed at defining modulatory effects of specific splice variants during the pathogenesis of sepsis and septic shock syndrome should reveal important new insights into the vascular endothelium's responses to LPS in particular and pathophysiology of sepsis syndrome in general.

## Supplementary Material

Supplementary file 1: Sequence alignment of splice variants of EGO and HOTAIRM1 lncRNA. Supplementary Table 1: Total reads from the RNA sequencing experiments at each treatment and percentage of reads mapped to coding (mRNA) and non-coding (ncRNA) transcripts. Supplementary Table 2: Expression level of regulatory (functionally annotated) splice variants of lncRNAs in 3 and 24 h LPS treated human microvascular endothelial cells, their genomic origins, and coding and non-coding indexes. Supplementary Table 3: Expression of Protein coding genes (PCGs) overlapping with functionally annotated lncRNAs at 3 and 24 h LPS treatment in human microvascular endothelial cells (HMECs). Supplementary Table 4: Significant canonical pathways and associated differentially regulated protein coding genes (PCGs) at 3 h in human microvascular endothelial cells (HMECs) after LPS treatment. Supplementary Table 5: Significant canonical pathways and associated differentially regulated protein coding genes (PCGs) at 24 h in human microvascular endothelial cells (HMECs) after LPS treatment.

## Figures and Tables

**Table 1:** List of primer sequences used for qRT-PCR.

lncRNA name	Primer name	Orientation	Sequence (5′-3′)
EGO (NONHSAT087634)	EGO-F	Forward	ACCCCAAACGAAAACAAGATAGAC
	EGO-R	Reverse	CCCATGCTAGCCAGCCTTTA
HOTAIRM1 (NONHSAT119666)	HOTAIRM1-F	Forward	GAAAGATGAACTGGCGAGAGAAA
	HOTAIRM1-R	Reverse	AGCTCCTGGATGCGATTCG
lnc-IL7R	lnc-IL7R-F	Forward	CCAGCCTTTGCCTCTTCCTTCAAT
	lnc-IL7R-R	Reverse	CCGTACCAAGTCTCT TAGCCC CTC

**Table 2:** Differentially expressed splice variants of functionally annotated lncRNAs from HMECs following LPS treatment for 3 and 24 h.

Functionally annotated lncRNAs	Total number of splice variants	Number of differentially expressed splice variants	Differentially expressed NONCODE splice variants	Class
ADAMTS9-AS2	11	2	NONHSAT090266, NONHSAT090274	Antisense

AK082072	39	9	NONHSAT102610, NONHSAT102619, NONHSAT102626	LINC
			NONHSAT102631, NONHSAT102632, NONHSAT102634	
			NONHSAT102637, NONHSAT102640, NONHSAT102641	

ANRIL	20	8	NONHSAT130413, NONHSAT130414, NONHSAT130416,	Antisense
			NONHSAT130417, NONHSAT130422, NONHSAT130423,	
			NONHSAT130425, NONHSAT130433	

CCAT1	16	1	NONHSAT129019	Antisense

CDR1-AS	5	1	NONHSAT138820	Antisense

CRNDE	13	2	NONHSAT142619, NONHSAT142620	LINC

CTBP1-AS	8	1	NONHSAT094692	LINC

CYP4A22-AS1	2	2	NONHSAT003050, NONHSAT003054	Antisense

Cyrano	14	2	NONHSAT041921, NONHSAT041926	Antisense

DHFR upstream transcripts	1	1	NONHSAT102417	Antisense; exonic

DHRS4-AS1	7	3	NONHSAT035952, NONHSAT035953, NONHSAT035955	Antisense

DISC2	2	1	NONHSAT010195	Exonic

DLEU1	48	6	NONHSAT033809, NONHSAT033813, NONHSAT033815	Antisense
			NONHSAT033822, NONHSAT033823, NONHSAT033850	

DLEU2	24	8	NONHSAT033771, NONHSAT033774, NONHSAT033780	Antisense
			NONHSAT033788, NONHSAT033796, NONHSAT033798	
			NONHSAT033800, NONHSAT033805	

EGO	3	2	NONHSAT087635, NONHSAT087634	Antisense

GAS5	33	3	NONHSAT007665, NONHSAT007698, NONHSAT007698	Antisense

Gomafu	52	6	NONHSAT084541, NONHSAT084545, NONHSAT084547	LINC
			NONHSAT084548, NONHSAT084549, NONHSAT084551	

H19	17	10	NONHSAT017460, NONHSAT017461, NONHSAT017461	LINC
			NONHSAT017465, NONHSAT017466, NONHSAT017467	
			NONHSAT017469, NONHSAT017471, NONHSAT017472,	
			NONHSAT017474	

HOTAIRM1	5	4	NONHSAT119664, NONHSAT119665, NONHSAT119666,	LINC
			NONHSAT119667	

Hoxa11as	9	1	NONHSAT119711	Antisense

IPW	81	22	NONHSAT041066, NONHSAT040981, NONHSAT040985	Antisense
			NONHSAT040986, NONHSAT040988, NONHSAT040989	
			NONHSAT040990, NONHSAT040991, NONHSAT041000	
			NONHSAT041004, NONHSAT041006, NONHSAT041023	
			NONHSAT041028, NONHSAT041047, NONHSAT041054	
			NONHSAT041057, NONHSAT041060, NONHSAT041061	
			NONHSAT041062, NONHSAT041064, NONHSAT041065	
			NONHSAT041140	

Jpx	14	3	NONHSAT137572, NONHSAT137582, NONHSAT137583	LINC

LINC00568	2	1	NONHSAT006301	LINC

LUCAT1	13	4	NONHSAT102744, NONHSAT102748, NONHSAT102749	LINC
			NONHSAT102750	

linc00467	6	6	NONHSAT009289, NONHSAT009290, NONHSAT009291	LINC
			NONHSAT009292, NONHSAT009293, NONHSAT009294	

lincRNA-SFMBT2	8	1	NONHSAT011261	LINC

HMECs: human dermal microvascular endothelial cells; LINC: long intergenic noncoding RNA.

**Table 3:** Comparison of lncRNAs differentially expressed during LPS treatment in HUVECs [22] and HMECs.

Differentially expressed lncRNAs in HUVECs (24 h)	Differentially expressed splice variants in HMECs (NONCODE IDs)	Fold change in HMECs (3 h)	Fold change in HMECs (24 h)
AL132709.5	NONHSAT039831	1.18	0.746
	NONHSAT039832	1.37	0.908
	NONHSAT039834	1.24	0.651
	NONHSAT039835	1.36	0.500
	NONHSAT039853	1.20	0.957
	NONHSAT039854	1.21	0.706
	NONHSAT039862	1.95	0.680
	NONHSAT039869	1.54	0.821
	NONHSAT140176	1.15	**4.095**

CLDN10-AS1	NONHSAT034761	**ID** ^∗^	**IU** ^∗^
	NONHSAT034762	**IU** ^∗^	**IU** ^∗^

AC068282.3	NONHSAT074154	Not detectable	Not detectable
	NONHSAT074148	1.26	1.026
	NONHSAT074149	1.78	0.339
	NONHSAT074150	0.96	1.140
	NONHSAT074151	0.72	2.047
	NONHSAT074152	0.58	1.143
	NONHSAT074153	1.81	**4.777**

RP11-534G20.3	NONHSAT012520	0.94	0.751
	NONHSAT012518	IU^∗^	0.350
	NONHSAT012519	**3.12**	1.370
	NONHSAT012522	ID^∗^	0.854
	NONHSAT012523	1.56	0.844
	NONHSAT012524	0.72	0.456
	NONHSAT012525	0.36	**ID** ^∗^
	NONHSAT012527	**IU** ^∗^	**IU** ^∗^
	NONHSAT012528	0.96	**IU** ^∗^
	NONHSAT012534	0.37	**−5.861**
	NONHSAT012521	2.75	1.090

XLOC_009994	NONHSAT026026	1.44	**IU** ^∗^
	NONHSAT026024	1.24	0.826
	NONHSAT026027	**IU** ^∗^	**IU** ^∗^
	NONHSAT140047	Not detectable	Not detectable

AC016683.6	NONHSAT073764	1.44	0.341
	NONHSAT073753	**3.60**	**ID** ^∗^
	NONHSAT073758	Not detectable	Not detectable
	NONHSAT073759	**ID** ^∗^	**IU** ^∗^
	NONHSAT073761	0.36	1.345
	NONHSAT073762	2.86	0.743
	NONHSAT073763	1.08	1.168
	NONHSAT073767	Not detectable	Not detectable
	NONHSAT073766	1.29	**IU** ^∗^
	NONHSAT073765	1.93	1.019
	NONHSAT073768	**4.13**	**3.194**
	NONHSAT073769	1.66	1.348
	NONHSAT073770	0.72	0.682
	NONHSAT073771	Not detectable	Not detectable
	NONHSAT073772	0.72	0.749
	NONHSAT073773	**0.24**	**IU** ^∗^

RP11-466I1.1	NONHSAT018559	Not detectable	Not detectable

RP11-184E9.2	NONHSAT100773	Not detectable	Not detectable

CTC-459I6.1	NONHSAT102422	Not detectable	Not detectable
	NONHSAT102423	Not detectable	Not detectable
	NONHSAT102425	Not detectable	Not detectable
	NONHSAT102426	Not detectable	Not detectable
	NONHSAT102427	Not detectable	Not detectable
	NONHSAT102428	Not detectable	Not detectable
	NONHSAT102424	Not detectable	Not detectable

RP11-138B4.1	NONHSAT099689	Not detectable	Not detectable
	NONHSAT099690	Not detectable	Not detectable

RP11-676J12.6	NONHSAT144771	Not detectable	Not detectable

XLOC_007697	NONHSAT131377	**0.29**	1.136

BX284650.1	NONHSAT005737	**IU** ^∗^	**ID** ^∗^
	NONHSAT005738	**ID** ^∗^	**IU** ^∗^
	NONHSAT005750	1.44	**IU** ^∗^
	NONHSAT005751	**0.07**	**IU** ^∗^
	NONHSAT005752	**IU** ^∗^	**IU** ^∗^
	NONHSAT005753	Not detectable	Not detectable
	NONHSAT005754	Not detectable	Not detectable
	NONHSAT005755	Not detectable	Not detectable

RP5-907D15.2	NONHSAT080552	**ID** ^∗^	**IU** ^∗^
	NONHSAT080546	0.68	0.488
	NONHSAT080547	0.36	**IU** ^∗^
	NONHSAT080548	Not detectable	Not detectable
	NONHSAT080549	Not detectable	Not detectable
	NONHSAT080551	Not detectable	Not detectable
	NONHSAT080550	0.72	0.682
	NONHSAT080553	Not detectable	Not detectable
	NONHSAT080555	Not detectable	Not detectable

XLOC_006311	NONHSAT124447	0.72	0.339

Note: up- or downregulation by ≥3-fold is indicated in bold. ID^∗^: infinite downregulation (no detectable reads after LPS treatment/presence of detectable reads in the controls). IU^∗^: infinite upregulation (presence of detectable reads following LPS treatment/no detectable reads in the controls).

**Table 4:** Significant canonical pathways identified by Ingenuity Pathway Analysis (IPA) in HMECs after 3 h treatment with LPS.

Ingenuity canonical pathways	−log(*P* value)
Granulocyte adhesion and diapedesis	4.1
Role of macrophages, fibroblasts, and endothelial cells in rheumatoid arthritis	3.9
Agranulocyte adhesion and diapedesis	3.7
Atherosclerosis signaling	3.7
Glucocorticoid receptor signaling	3.5
Role of IL-17A in arthritis	3.5
Activation of IRF by cytosolic pattern recognition receptors	3.5
Role of hypercytokinemia/hyperchemokinemia in the pathogenesis of influenza	3.3
IL-17 signaling	3.2
Role of IL-17A in psoriasis	3.2
Death receptor signaling	3.1
HMGB1 signaling	3.0
Hepatic fibrosis/hepatic stellate cell activation	3.0
Differential regulation of cytokine production in macrophages and T helper cells by IL-17A and IL-17F	2.9
TNFR2 signaling	2.9
TREM1 signaling	2.7
Differential regulation of cytokine production in intestinal epithelial cells by IL-17A and IL-17F	2.7
Communication between innate and adaptive immune cells	2.6
Acute-phase response signaling	2.5
Role of cytokines in mediating communication between immune cells	2.4
Role of IL-17F in allergic inflammatory airway diseases	2.4
TNFR1 signaling	2.4
Dendritic cell maturation	2.2
IL-17A signaling in gastric cells	2.1
IL-17A signaling in airway cells	2.0
Inflammasome pathway	2.0
Role of pattern recognition receptors in recognition of bacteria and viruses	2.0
IL-6 signaling	1.9
TWEAK signaling	1.8
Systemic lupus erythematosus signaling	1.8
CD40 signaling	1.7
Hepatic cholestasis	1.6
LXR/RXR activation	1.6
Retinoic acid-mediated apoptosis signaling	1.6
Lymphotoxin *β* receptor signaling	1.6
IL-17A signaling in fibroblasts	1.6
PPAR signaling	1.6
Eicosanoid signaling	1.6
Hematopoiesis from pluripotent stem cells	1.5
Altered T cell and B cell signaling in rheumatoid arthritis	1.4
Coagulation system	1.3
B cell-activating factor signaling	1.3
Role of osteoblasts, osteoclasts, and chondrocytes in rheumatoid arthritis	1.3
Induction of apoptosis by HIV1	1.3
Cholecystokinin/gastrin-mediated signaling	1.3
PI3K signaling in B lymphocytes	1.3
Toll-like receptor signaling	1.3

**Table 5:** Significant canonical pathways identified by Ingenuity Pathway Analysis (IPA) in HMECs after 24 h treatment with LPS.

Ingenuity canonical pathways	−log(*P* value)
Role of IL-17F in allergic inflammatory airway diseases	7.1
Role of IL-17A in arthritis	5.7
Agranulocyte adhesion and diapedesis	5.7
Granulocyte adhesion and diapedesis	5.4
Role of IL-17A in psoriasis	4.9
Role of hypercytokinemia/hyperchemokinemia in the pathogenesis of influenza	4.6
Role of macrophages, fibroblasts, and endothelial cells in rheumatoid arthritis	4.6
Communication between innate and adaptive immune cells	4.5
Differential regulation of cytokine production in macrophages and T helper cells by IL-17A and IL-17F	4.3
HMGB1 signaling	4.2
Differential regulation of cytokine production in intestinal epithelial cells by IL-17A and IL-17F	4.0
Role of cytokines in mediating communication between immune cells	3.5
Glucocorticoid receptor signaling	3.5
Atherosclerosis signaling	3.4
Retinol biosynthesis	3.4
Hepatic fibrosis/hepatic stellate cell activation	3.0
Acute-phase response signaling	2.9
IL-17 signaling	2.8
Graft-versus-host disease signaling	2.8
IL-17A signaling in fibroblasts	2.6
Hematopoiesis from pluripotent stem cells	2.5
Dendritic cell maturation	2.5
TREM1 signaling	2.4
IL-17A signaling in gastric cells	2.3
IL-17A signaling in airway cells	2.3
The visual cycle	2.3
Role of pattern recognition receptors in recognition of bacteria and viruses	2.2
Altered T cell and B cell signaling in rheumatoid arthritis	2.2
Role of osteoblasts, osteoclasts, and chondrocytes in rheumatoid arthritis	2.0
Systemic lupus erythematosus signaling	2.0
Activation of IRF by cytosolic pattern recognition receptors	1.9
Role of MAPK signaling in the pathogenesis of influenza	1.9
Hepatic cholestasis	1.9
LXR/RXR activation	1.9
Retinoate biosynthesis I	1.8
Bile acid biosynthesis, neutral pathway	1.8
Methylglyoxal degradation III	1.8
Airway pathology in chronic obstructive pulmonary disease	1.8
IL-6 signaling	1.6
Crosstalk between dendritic cells and natural killer cells	1.5
Triacylglycerol degradation	1.5
Induction of apoptosis by HIV1	1.5
Cholecystokinin/gastrin-mediated signaling	1.5
OX40 signaling pathway	1.5
Chemokine signaling	1.4
Role of tissue factor in cancer	1.4
Antigen presentation pathway	1.3
PPAR signaling	1.3
