# Supplementary material for: Expression Profiling of Long Noncoding RNA Splice Variants in Human Microvascular Endothelial Cells: Lipopolysaccharide Effects In Vitro
Source: Mediators Inflamm. 2017 Sep 25;2017:3427461. doi: 10.1155/2017/3427461 (PMC5632992; doi:10.1155/2017/3427461)
Supplement: Supplementary file 1 — Sequence alignment of splice variants of EGO and HOTAIRM1 lncRNA. Supplementary Table 1: Total reads from the RNA sequencing experiments at each treatment and percentage of reads mapped to coding (mRNA) and non-coding (ncRNA) transcripts. Supplementary Table 2: Expression level of regulatory (functionally annotated) splice variants of lncRNAs in 3 and 24 h LPS treated human microvascular endothelial cells, their genomic origins, and coding and non-coding indexes. Supplementary Table 3: Expression of Protein coding genes (PCGs) overlapping with functionally annotated lncRNAs at 3 and 24 h LPS treatment in human microvascular endothelial cells (HMECs). Supplementary Table 4: Significant canonical pathways and associated differentially regulated protein coding genes (PCGs) at 3 h in human microvascular endothelial cells (HMECs) after LPS treatment. Supplementary Table 5: Significant canonical pathways and associated differentially regulated protein coding genes (PCGs) at 24 h in human microvascular endothelial cells (HMECs) after LPS treatment. [file 3427461.f1.pdf]

Supplementary file 1: Sequence alignment of splice variants of EGO and HOTAIRM1 lncRNA.

<u>CLUSTAL O (1.2.1) multiple sequence alignment of overlapping splice variants of EGO</u>

```
NONHSAT087636      ------------------------------------------------------------
NONHSAT087634      ----------------CAGAGGTGGGTTTGGCTTTTTAAAAATTTTTTCAGCCTGTCCTC
NONHSAT087635      CAACTTCTGGGCAAGGCAGAGGTGGGTTTGGCTTTTTAAAAATTTTTTCAGCCTGTCCTC


NONHSAT087636      ------------------------------------------------------------
NONHSAT087634      ATGGAACTACATATTCTTTTCTAAGAACTTTTCATCCTAACCTCCCTACTCACATCTTCT
NONHSAT087635      ATGGAACTACATATTCTTTTCTAAGAACTTTTCATCCTAACCTCCCTACTCACATCTTCT


NONHSAT087636      ------------------------------------------------------------
NONHSAT087634      AAGTGTCTCTGCTCTGGTGGGAATGTGATGGACAACACAGAGCCATCTCAGAAGCCTCTG
NONHSAT087635      AAGTGTCTCTGCTCTGGTGGGAATGTGATGGACAACACAGAGCCATCTCAGAAGCCTCTG


NONHSAT087636      ------------------------------------------------------------
NONHSAT087634      TGGCCACCACCAGGCCGGCCAGGGTGCAGGGGGCCACTCCCTGGGCAGCCATAGGGTTCT
NONHSAT087635      TGGCCACCACCAGGCCGGCCAGGGTGCAGGGGGCCACTCCCTGGGCAGCCATAGGGTTCT


NONHSAT087636      ----------------------------------------------------GAAGTTAA
NONHSAT087634      CAGCAAGGTGCATTCGTCGTCCCTGCTGAGAATCTGATGGGGCAGCATTTTTTTTTTTAA
NONHSAT087635      CAGCAAGGTGCATTCGTCGTCCCTGCTGAGAATCTGATGGGGCAGCATTTTTTTTTTTAA
                                                                       ****

NONHSAT087636      T-AAAGGG-----AAGTC-----AGCTTGCC----TCTG-----ATTATTCAAACAGGCT
NONHSAT087634      TTAAATGCAAGCTGAGTCATTTCAACCTGCAACCTTCAGGTAACAGGAGTTACCCAAGCT
NONHSAT087635      TTAAATGCAAGCTGAGTCATTTCAACCTGCAACCTTCAGGTAACAGGAGTTACCCAAGCT
                   * *** *       ****     * * ***     ** *     *  * * *  ** ***

NONHSAT087636      ACTGCTATTAAG------G----------------------ATTGCACAACTTCTGGGCA
NONHSAT087634      CCAGGAATTATGATTGTGGGGTAAACCCATTCTCTTGTTTTCTTGCGGTTCTATTTTATA
NONHSAT087635      CCAGGAATTATGATTGTGGGGTAAACCCATTCTCTTGTTTTCTTGCGGTTCTATTTTATA
                    * *  **** *      *                       ****    **  *    *

NONHSAT087636      AGG-CAGAGAGGAGACAGAGACGTCATTGCTTCACCCAGGGCAAGAGGACAAAGGAGGAT
NONHSAT087634      ACGCACTAGAGGAGACAGAGACGTCATTGCTTCACCCAGGGCAAGAGGACAAAGGAGGAT
NONHSAT087635      ACGCACTAGAGGAGACAGAGACGTCATTGCTTCACCCAGGGCAAGAGGACAAAGGAGGAT
                   * *    *****************************************************

NONHSAT087636      GCATGGAAATACAAACAGCCCTTCTCCTCCAGGCCATACCGACTGTCCAACTAGCAACAG
NONHSAT087634      GCATGGAAATACAAACAGCCCTTCTCCTCCAGGCCATACCGACTGTCCAACTAGCAACAG
NONHSAT087635      GCATGGAAATACAAACAGCCCTTCTCCTCCAGGCCATACCGACTGTCCAACTAGCAACAG
                   ************************************************************

NONHSAT087636      ACTTCACCTGGTTTTGGAGCAATCTGAATTTGGAATATGCCAGAGAAAACTTCTATCAGG
NONHSAT087634      ACTTCACCTGGTTTTGGAGCAATCTGAATTTGGAATATGCCAGAGAAAACTTCTATCAGG
NONHSAT087635      ACTTCACCTGGTTTTGGAGCAATCTGAATTTGGAATATGCCAGAGAAAACTTCTATCAGG
                   ************************************************************
```

```
NONHSAT087636       CAAGATGGAAGACTCCTAGGATAGGTCTCTCTCATGGAGGGATTGGTAATAAA---AAAA
NONHSAT087634       C----------------------------------------------ACGATGGTAAGAG
NONHSAT087635       CAAGATGGAAGACTCCTAGGATAGGTCTCTCTCATGGAGGGATTGGCACGATGGTAAGAG
                    *                                              *  *     * *

NONHSAT087636       TCAT-----GGGAAAAGAAA------TAAGGGAGGG---TGGAAA-AAACACACCTTAGA
NONHSAT087634       TCCAGTGGATGGAAAAGGCCTCCTCCTTAAAGATGGTGATGGAAACGAGCACTGCACAGG
NONHSAT087635       TCCAGTGGATGGAAAAGGCCTCCTCCTTAAAGATGGTGATGGAAACGAGCACTGCACAGG
                    **        *******         * *  ** **   ******  * ***  *  **

NONHSAT087636       AAACAACTTCTGATGTCCCGGG-----------------GCTACAC------------AA
NONHSAT087634       GAAACACAAATCAGGTCCCCTGAAATCCAGCCCAACCTGGCCAGACCCTCCAGTGCCCAT
NONHSAT087635       GAAACACAAATCAGGTCCCCTGAAATCCAGCCCAACCTGGCCAGACCCTCCAGTGCCCAT
                     **  **    * * *****  *                 ** * **            *

NONHSAT087636       TGGGGGAGCTGAGC----------------------------------------------
NONHSAT087634       CAGGGCTTATGAACAGGGTGCTTCAGTGTCTTTGTTAGGGGTGGTTAAAAAGGAGCACGT
NONHSAT087635       CAGGGCTTATGAACAGGGTGCTTCAGTGTCTTTGTTAGGGGTGGTTAAAAAGGAGCACGT
                      ***    *** *

NONHSAT087636       ------------------------------------------------------------
NONHSAT087634       GCTTATAGGGGATGCTGCTGAGCTCCATGATTTTGACTTCCTGTCTAACCTGTTGATGCT
NONHSAT087635       GCTTATAGGGGATGCTGCTGAGCTCCATGATTTTGACTTCCTGTCTAACCTGTTGATGCT

NONHSAT087636       ------------------------------------------------------------
NONHSAT087634       ACAAAACTCTTTTAAAAACAGTTACCATGGGAACTTTTCCCTAGCAAGCATTCTTCAAGA
NONHSAT087635       ACAAAACTCTTTTAAAAACAGTTACCATGGGAACTTTTCCCTAGCAAGCATTCTTCAAGA

NONHSAT087636       ------------------------------------------------------------
NONHSAT087634       AACGTTACAGGGAGCTGTTGTTGGGACAGGACAAGAGTCCACATCCCCTG---CCCAGTG
NONHSAT087635       AACGTTACAGGTG-CTGGTG---------------AATTCAATGCCCTTGGTGTCAATAA

NONHSAT087636       ------------------------------------------------------------
NONHSAT087634       CCTGGTTCTGGGTCATGTCACCGAAGCGCATCAGCCCATCTGTCAGCCAAACCCCACTAG
NONHSAT087635       GCTTTTGCTGAGTCTTAGA------------------ATG--------------------

NONHSAT087636       ------------------------------------------------------------
NONHSAT087634       CCTTGTGCCTTCTCTTCTAAATCCCCGCGGAGGA-----AAACAACTTCTGCC--TTCTA
NONHSAT087635       ---TGTGGCTTGAAC-----------TGAGAGGACTTTCAAACAGATAATGCGAAGTTTC

NONHSAT087636       ------------------------------------------------------------
NONHSAT087634       CAGTACGAACAGCGAGGGTCTGCTGGGAACCAATACAACTTCGCGTCCCAGTGCAGCAAT
NONHSAT087635       CAA-------TGCAAATGTCTTCGGGG-------------TTAAGTGTCAGTGAAGTGCA

NONHSAT087636       ------------------------------------------------------------
NONHSAT087634       GTTGCCAGGTAACAAATGTAGAAACATAC--TCCAAAAACTGTATGTGACAATATACAAC
NONHSAT087635       GT-GGAAGG---------TAGGGGGATAAAATGATCTCAGTGTA--------TATGGAGG

NONHSAT087636       ------------------------------------------------------------
NONHSAT087634       CTCCGCTCTGCAGCTGATAACCGAGGGAGTGTTAAAACACCGCGTTCTCTTAACACTTGG
NONHSAT087635       CCCCA--CTTAAGC----------------ATTCAAAAA----ATTCTATATACAGT---
```

```
NONHSAT087636      ------------------------------------------------------------
NONHSAT087634      GTTTTTG-------GTGTGAAAGTAAAATTAAACGGGATACTTCCTCTTGTAAATATAAG
NONHSAT087635      GTTTTCAATGTATTATGTATCAGCAAAACAA-----AATACCC----------------C

NONHSAT087636      ------------------------------------------------------------
NONHSAT087634      ACAGGCATACAACTCACACACGTATCCACTAATCGGAAATAGTAGAGGGGTTTGCAATAG
NONHSAT087635      ATAGGGCTATAA--------------------TC------AGCTCAGGGGGTCACCTCAG

NONHSAT087636      ------------------------------------------------------------
NONHSAT087634      CTAT--CTGCAGTGCATCCCAGCCTTCACTCTTACCCTCAAGATGAATTCCAGAAAACCT
NONHSAT087635      CAAGAGATGCT-------CTGGCTATCACTCCTACT------------------------


NONHSAT087636      ------------------------------------------------------------
NONHSAT087634      ACAGAAACGATAACTGAGGAAGAGCCTTTCTTCTGTCGGGGACAAGCTCTCCTCTAACAA
NONHSAT087635      ---------------ACGG-----GATTTCCTCTCTC----------------ATCAGAA

NONHSAT087636      ------------------------------------------------------------
NONHSAT087634      TTGTGAGGAAG--CTACCCACAAGAAAGACACCAGCAGAAATTAAG-----GAAAAGCTC
NONHSAT087635      CCAGTAGGTGGTCAAAACCACAATAA-------TGCATAAATGAAGGCAGGGAATTGGGT

NONHSAT087636      ------------------------------------------------------------
NONHSAT087634      ATGCATTGTTATTAAAAAAAAATGTGCTTCCATTCCACTCCATTTTAAAGGTCTACCTTT
NONHSAT087635      ATCCAATGTGAGCAACTGAAAAGGTGCCATGT-----------ATTAGCTATTTGCCATT

NONHSAT087636      ------------------------------------------------------------
NONHSAT087634      AAAACTCATTGGCTAACCAAGCAGTGACTACTTGCTTCTCAGATCAAATAACCAGTGCAG
NONHSAT087635      A--------TGGTTCTGCAAG---TG----CTCAGATGACTCATAAAATGACCAGAACAA

NONHSAT087636      ------------------------------------------------------------
NONHSAT087634      CCTGTTCCCACCCCAAACGAAAACAAGATAGACACTGCTCTCTAGTAGGCAGAGCTCCAA
NONHSAT087635      AA----------------------------------------------------------

NONHSAT087636      ------------------------------------------------------------
NONHSAT087634      AGTGGGTACAGGGCACTGAATAGCTCCCTCACATTGTACTCATGTAACTTAAAGGCTGGC
NONHSAT087635      ------------------------------------------------------------

NONHSAT087636      ------------------------------------------------------  406
NONHSAT087634      TAgcatgggagaaagctgagagacttctgagggccttgtgttcaaatcccagct  1824
NONHSAT087635      ------------------------------------------------------  1464
```

CLUSTAL O (1.2.1) multiple sequence alignment of overlapping splice variants of HOTAIRM1

```
NONHSAT119666      ------------------------------------------------------------
NONHSAT119667      ------------------------------------------------------------
NONHSAT119668      ------------------------------------------------------------
NONHSAT119664      -------------------------AAAAGTTTGCCGGCTTCCGCAGTGATGGATCACCG
NONHSAT119665      -------------------------AAAAGTTTGCCGGCTTCCGCAGTGATGGATCACCG
```

NONHSAT119666 --
NONHSAT119667 --
NONHSAT119668 --
NONHSAT119664 TTTTAGTGGCATTTAAATCCCCGGCGCTCCGCCGTCTAGGTGACGCGCAGTCGCCCCCCC
NONHSAT119665 TTTTAGTGGCATTTAAATCCCCGGCGCTCCGCCGTCTAGGTGACGCGCAGTCGCCCCCCC

NONHSAT119666 --GATTTGGAGTGCTGGA
NONHSAT119667 --
NONHSAT119668 --
NONHSAT119664 AGGCAGCCTAGGCGGCGGCAGCTGCTGCGGCGACTGCAAAGGCCGATTTGGAGTGCTGGA
NONHSAT119665 AGGCAGCCTAGGCGGCGGCAGCTGCTGCGGCGACTGCAAAGGCCGATTTGGAGTGCTGGA

NONHSAT119666 GCGAAGAAGAGCAAAAGCTGCGTTCTGCGCGCGCCCGACTCCGCTGCCCGCCCCGCCAGG
NONHSAT119667 --
NONHSAT119668 --
NONHSAT119664 GCGAAGAAGAGCAAAAGCTGCGTTCTGCGCGCGCCCGACTCCGCTGCCCGCCCCGCCAGG
NONHSAT119665 GCGAAGAAGAGCAAAAGCTGCGTTCTGCGCGCGCCCGACTCCGCTGCCCGCCCCGCCAGG

NONHSAT119666 CCTCCGGGAGGTGGGGGCTGGGAGGCGTCCCCCGCTCCCGCCCCCTCCCCACCGTTCAAT
NONHSAT119667 --
NONHSAT119668 --
NONHSAT119664 CCTCCGGGAGGTGGGGGCTGGGAGGCGTCCCCCGCTCCCGCCCCCTCCCCACCGTTCAAT
NONHSAT119665 CCTCCGGGAGGTGGGGGCTGGGAGGCGTCCCCCGCTCCCGCCCCCTCCCCACCGTTCAAT

NONHSAT119666 **GAAAGATGAACTGGCGAGA**---
NONHSAT119667 --------------------TGCTAGAAAGGAAAGGAATAAGGAGGAGATAAAATGACGC
NONHSAT119668 --
NONHSAT119664 GAAAGATGAACTGGCGAGAG--
NONHSAT119665 GAAAGATGAACTGGCGAGAG--

NONHSAT119666 --
NONHSAT119667 CGAGGCCCTGAACTGTTCATGGCATCCGCGGCTCAGCCAAGCTGttgttttaaaagagca
NONHSAT119668 --
NONHSAT119664 --
NONHSAT119665 --

NONHSAT119666 --
NONHSAT119667 ataaaaatgaattatgactaaacgccttctaacttaatgctttcggacggggatccccgg
NONHSAT119668 --
NONHSAT119664 --
NONHSAT119665 --

NONHSAT119666 --
NONHSAT119667 caaaTAACGTAAGAGGATTTTTATTTGTGCATGTGTTCCTGCAATTGATCTCTTTGATGA
NONHSAT119668 --
NONHSAT119664 --
NONHSAT119665 --

NONHSAT119666 --------------**GAAA**GCGTTTGATTTATGAGCGTAGGA**CGAATCGCATCCAGGAGCT**
NONHSAT119667 CATTCTCATTCATAGAAAGCGTTTGATTTATGAGCGTAGGACGAATCGCATCCAGGAGCT
NONHSAT119668 --
NONHSAT119664 ---------------------------------------GACGAATCGCATCCAGGAGCT
NONHSAT119665 --

```
NONHSAT119666      GCGCAGCCCTGGCCGCTGCCGGGACGCCCTGCTCCGCGCTGAGCTTGGGGCCAGAAACCA
NONHSAT119667      GCGCAGCCCTGGCCGCTGCCGGGACGCCCTGCTCCGCGCTGAGCTTGGGGCCAGAAACCA
NONHSAT119668      -------------------------------------------------------AAACA
NONHSAT119664      GCGCAGCCCTGGCCGCTGCCGGGACGCCCTGCTCCGCGCTGAGCTTGGGGCCAGAAACCA
NONHSAT119665      ------------------------------------------------------------

NONHSAT119666      GCCATAGTCCCCACACTCCGCCGCCGCAGCTGAGATTTAGCGGAGGAAGGGGCGAGGGAA
NONHSAT119667      GCCATAGTCCCCACACTCCGCCGCCGCAGCTGAGATTTAGCGGAGGAAGGGGCGAGGGAA
NONHSAT119668      GGTTTAAGAATGA--------------AATATTGATTTTTCTGACCC--------TGAA-
NONHSAT119664      GCCATAGTCCCCACACTCCGCCGCCGCAGCTGAGATTTAGCGGAGGAAGGGGCGAGGGAA
NONHSAT119665      ------------------------------------------------------------

NONHSAT119666      GGTAGGGAGCAAACCTATGAAGAAACATCGCGTTGTCA-------------TTGGAACTT
NONHSAT119667      GGTAGGGAGCAAACCTATGAAGAAACATCGCGTTGTCA-------------TTGGAACTT
NONHSAT119668      -----------AA---AAAAAAAAAAATCCTAGAGTGACTGAACCAGAGGATTGGAAAGA
NONHSAT119664      GGTAGGGAGCAAACCTATGAAGAAACATCGCGTTGTCA-------------TTGGAACTT
NONHSAT119665      ------------------------------------------------------------

NONHSAT119666      CCAAGCCTTTGCTGTTAAGAGCCAGGTTCTTAAATCAACCCGCCCCACACACATGTTGCT
NONHSAT119667      CCAAGCCTTTGCTGTTAAGAGCCAGGTTCTTAAATCAACCCGCCCCACACACATGTTGCT
NONHSAT119668      ----ACATTTTCT---AGGGTGCATTTTCCA-GAT--------AGTAATCActgttttgt
NONHSAT119664      CCAAGCCTTTGCTGTTAAGAGCCAGGTTCTTAAATCAACCCGCCCCACACACATGTTGCT
NONHSAT119665      ------------------------------------------------------------

NONHSAT119666      TACATGCTGCGTTTTC-------TCACGGTCTGTTTTGCCTGAACCCATCAACAGCTGGG
NONHSAT119667      TACATGCTGCGTTTTC-------TCACGGTCTGTTTTGCCTGAACCCATCAACAGCTGGG
NONHSAT119668      tttatttttgtttttctctatcttttAGGTCTGTTTTGCCTGAACCCATCAACAGCTGGG
NONHSAT119664      TACATGCTGCGTTTTC-------TCACGGTCTGTTTTGCCTGAACCCATCAACAGCTGGG
NONHSAT119665      ----------------------------GTCTGTTTTGCCTGAACCCATCAACAGCTGGG
                                               ********************************

NONHSAT119666      AGATTAATCAACCACACTGAAAATGTGGAGGGATTTATGGGGGAGGGGGTTGAAATGTGG
NONHSAT119667      AGATTAATCAACCACACTGAAAATGTGGAGGGATTTATGGGGGAGGGGGTTGAAATGTGG
NONHSAT119668      AGATTAATCAACCACACTGAAAATGTGGAGGGATTTATGGGGGAGGGGGTTGAAATGTGG
NONHSAT119664      AGATTAATCAACCACACTGAAAATGTGGAGGGATTTATGGGGGAGGGGGTTGAAATGTGG
NONHSAT119665      AGATTAATCAACCACACTGAAAATGTGGAGGGATTTATGGGGGAGGGGGTTGAAATGTGG
                   ************************************************************

NONHSAT119666      GTGTTTGAAACAAAAGTGTATAAACAAATGAATTGTTGATAACTTAGTTATTGACCTGGA
NONHSAT119667      GTGTTTGAAACAAAAGTGTATAAACAAATGAATTGTTGATAACTTAGTTATTGACCTGGA
NONHSAT119668      GTGTTTGAAACAAAAGTGTATAAACAAATGAATTGTTGATAACTTAGTTATTGACCTGGA
NONHSAT119664      GTGTTTGAAACAAAAGTGTATAAACAAATGAATTGTTGATAACTTAGTTATTGACCTGGA
NONHSAT119665      GTGTTTGAAACAAAAGTGTATAAACAAATGAATTGTTGATAACTTAGTTATTGACCTGGA
                   ************************************************************

NONHSAT119666      GACTGGTAGCTTATTAAAGAAACTCCGTGTTACTCA------------------------
NONHSAT119667      GACTGGTAGCTTATTAAAGAAACTCCGTGTTACTCATTCCTGGAGTTGGGGGTTTCTGTA
NONHSAT119668      GACTGGTAGCTTATTAAAGAAACTCCGTGTTACTCATTCCTGGAGTTGGGGGTTTCTGTA
NONHSAT119664      GACTGGTAGCTTATTAAAGAAACTCCGTGTTACTCATTCCTGGAGTTGGGGGTTTCTGTA
NONHSAT119665      GACTGGTAGCTTATTAAAGAAACTCCGTGTTACTCATTCCTGGAGTTGGGGGTTTCTGTA
                   ************************************

NONHSAT119666      ------------------------------------------------------------
NONHSAT119667      GGCACTTTATTTCTCCACTTTCAAGAGCTTGGGCTTGGCCCAAATCTTAGACTGTCCAAT
NONHSAT119668      GGCACTTTATTTCTCCACTTTCAAGAGCTTGGGCTTGGCCCAAATCTTAGACTGTCCAAT
NONHSAT119664      GGCACTTTATTTCTCCACTTTCAAGAGCTTGGGCTTGGCCCAAATCTTAGACTGTCCAAT
NONHSAT119665      GGCACTTTATTTCTCCACTTTCAAGAGCTTGGGCTTGGCCCAAATCTTAGACTGTCCAAT
```

```
NONHSAT119666      ------------------------------------------------------------
NONHSAT119667      TCTGCCTCTATTACCAATTTAAATCTATGGCTTGAACCTGTGCACTGAAAATCAAATCCT
NONHSAT119668      TCTGCCTCTATTACCAATTTAAATCTATGGCTTGAACCTGTGCACTGAAAATCAAATCCT
NONHSAT119664      TCTGCCTCTATTACCAATTTAAATCTATGGCTTGAACCTGTGCACTGAAAATCAAATCCT
NONHSAT119665      TCTGCCTCTATTACCAATTTAAATCTATGGCTTGAACCTGTGCACTGAAAATCAAATCCT

NONHSAT119666      ------------------------------------------------------------
NONHSAT119667      TTaaaaagaaagaggagaagaagaagcaa-------------------------------
NONHSAT119668      TTaaaaagaaagaggagaagaagaagcaaaaaaagaaagaaaaaACACTTATTAGAAGCCC
NONHSAT119664      TTaaaaagaaagaggagaagaagaagcaaaaaaagaaagaaaaaACACTTATTAGAAGCCC
NONHSAT119665      TTaaaaagaaagaggagaagaagaagcaaaaaaagaaagaaaaaACACTTATTAGAAGCCC

NONHSAT119666      ------------------------------------------------------------
NONHSAT119667      ------------------------------------------------------------
NONHSAT119668      TAGTCATTTTTTGGCTTTCTGTTTTGTTGCTGTCCATTGAAGACTTTGAACATGCCGCCT
NONHSAT119664      TAGTCATTTTTTGGCTTTCTGTTTTGTTGCTGTCCATTGAAGACTTTGAACATGCCGCCT
NONHSAT119665      TAGTCATTTTTTGGCTTTCTGTTTTGTTGCTGTCCATTGAAGACTTTGAACATGCCGCCT

NONHSAT119666      ------------------------------------
NONHSAT119667      ------------------------------------
NONHSAT119668      TAATAAATGTATTAAAATTGAAAAAAGAGGATAAA
NONHSAT119664      TAATAAATGTATTAAAATTGAAAAAAGA-------
NONHSAT119665      TAATAAATGTATTAAAATTGAAAAAAGA-------
```

Supplementary table 1: Total reads from the RNA sequencing experiments at each treatment and percentage of reads mapped to coding (mRNA) and non-coding (ncRNA) transcripts.

Experiment	Total reads (in millions)	Reads mapped to mRNA (in millions)	Reads mapped to ncRNA (in millions)
3 h control	29.31	9.11 (31.08%)	3.26 (11.11%)
3 h LPS treatment	28.35	14.27 (37.87%)	4.52 (12%)
24 h control	37.68	9.2 (32.45%)	3.56 (12.57%)
24 h LPS treatment	34.86	12.99 (36.26%)	5.22 (14.98%)

Supplementary Table 2: Expression level of regulatory (functionally annotated) splice variants of lncRNAs in 3 and 24 h LPS treated human microvascular endothelial cells, their genomic origins, and coding and non-coding indexes.

Traditional IDs	Regulatory NONCODE splice variants	3h (Fold change)	24h (Fold change)	Chromosome Number	Transcription Start site	Transcription End site	Strand	Exon Number	Length	CNCI Score	References
ADAMTS9-AS2	NONHSAT090266	2.289	3.307	3	64670803	64997143	Positive	6	1969	-0.036	[1]
	NONHSAT090274	0.079	2.270		64949871	64964665		1	14794	-0.112	
AK082072	NONHSAT102610	0.720	ID	5	87564698	87732491	Positive	3	608	-0.077	[2]
	NONHSAT102619	0.726	0.113		87564863	87583473		7	717	-0.145	
	NONHSAT102626	0.103	0.509		87577500	87587245		5	881	-0.088	
	NONHSAT102631	0.308	0.694		87578275	87582807		2	1875	-0.016	
	NONHSAT102632	0.144	1.365		87578275	87587291		3	686	-0.267	
	NONHSAT102634	0.144	IU		87578328	87581854		2	649	-0.122	
	NONHSAT102637	0.301	0.632		87578532	87584540		3	1547	-0.133	
	NONHSAT102640	0.329	0.273		87582902	87587291		2	628	-0.195	
	NONHSAT102641	0.315	0.538		87588449	87589492		2	1043	-0.262	
ANRIL	NONHSAT130413	0.480	IU	9	21994776	22032955	Positive	4	631	-0.102	[3]
	NONHSAT130414	1.081	IU		21994789	22077889		4	856	-0.347	
	NONHSAT130416	1.294	0.200		21994789	22077889		6	1067	-0.196	
	NONHSAT130417	1.441	0.336		21994789	22077889		8	1260	-0.347	
	NONHSAT130422	0.180	1.372		21994789	22121093		7	1646	-0.347	
	NONHSAT130423	2.111	4.777		21994789	22121093		8	1782	-0.347	
	NONHSAT130425	0.295	0.456		21994789	22121093		6	1582	-0.078	
	NONHSAT130433	0.235	0.476		22111739	22128078		4	9091	-0.212	

CCAT1	NONHSAT129019	0.302	1.124	8	128098507	128099755	Negative	1	1248	-0.142	[4]
CDR1-AS	NONHSAT138820	ID	ID	X	139865228	139866833	Positive	3	923	-0.073	[5]
CRNDE	NONHSAT142619	1.422	IU	16	54957192	54962798	Negative	3	600	-0.432	[6]
	NONHSAT142620	ID	0.682		54958770	54963061		2	735	-0.157	
CTBP1-AS	NONHSAT094692	0.273	1.123	4	1244046	1249698	Positive	1	5652	-0.032	[7]
CYP4A22-AS1	NONHSAT003050	Not detectable	IU	1	47562324	47644943	Negative	4	921	-0.079	[8]
	NONHSAT003054	ID	IU		47638526	47640990		2	947	-0.249	
Cyrano	NONHSAT041921	ID	2.608	15	41576183	4159872	Positive	6	8797	-0.074	[9]
	NONHSAT041926	0.963	0.294		41576207	41577887		2	566	-0.268	
DHFR-upstream-transcripts	NONHSAT102417	ID	ID	5	79950178	79950765	Positive	1	587	-0.337	[10]
DHRS4-AS1	NONHSAT035952	Not detectable	ID	14	24407939	24423392	Negative	3	2747	-0.204	[11]
	NONHSAT035953	0.196	IU		24407939	24424298		2	2770	-0.054	
	NONHSAT035955	ID	1.024		24407939	24423406		2	2852	-0.198	
DISC2	NONHSAT010195	0.047	0.531	1	231948705	231956006	Positive	2	7289	-0.095	[12]
DLEU1	NONHSAT033809	IU	ID	13	50656306	50964427	Positive	2	481	-0.043	[13]
	NONHSAT033813	ID	Not detectable		50656306	51102670		6	1141	-0.12	
	NONHSAT033815	IU	Not detectable		50656306	51102200		6	982	-0.087	
	NONHSAT033822	IU	Not detectable		50656333	50679415		3	991	-0.386	
	NONHSAT033823	Not detectable	ID								
	NONHSAT033850	Not detectable	IU								

DLEU2	NONHSAT033771	0.326	0.481	13	50526410	50570895	Negative	3	22622	-0.086	[14]
	NONHSAT033774	ID	Not detectable		50530842	50562398		3	409	-0.173	
	NONHSAT033780	0.096	0.171		50557996	50561191		5	1711	-0.378	
	NONHSAT033788	4.322	0.409		50601279	50603690		2	540	-0.099	
	NONHSAT033796	IU	IU		50618522	50656128		4	699	-0.284	
	NONHSAT033798	2.166	0.273		50618650	50656108		6	705	-0.058	
	NONHSAT033800	ID	0.346		50623108	50623337		1	229	-0.628	
	NONHSAT033805	0.103	0.607		50623325	50656114		4	791	-0.058	
EGO	NONHSAT087635	1.409	IU	3	4790875	4793274	Negative	2	1464	-0.071	[15]
	NONHSAT087634	5.779	0.682		4789857	4793258		3	1824	-0.074	
GAS5	NONHSAT007665	IU	0.682	1	173833038	173837129		12	660	-0.134	[16]
	NONHSAT007698	1.435	4.100		173834955	173837125		3	1060	-0.17	
	NONHSAT007699	0.181	0.976								
Gomafu	NONHSAT084541	0.240	0.455	22	27068767	27174598	Positive	5	760	-0.226	[17]
	NONHSAT084545	ID	1.365		27089720	27176138		5	691	-0.037	
	NONHSAT084547	ID	ID		27113331	27118135		3	706	-0.108	
	NONHSAT084548	1.446	ID		27122619	27174541		4	451	-0.024	
	NONHSAT084549	0.412	ID		27130460	27134094		2	717	-0.458	
	NONHSAT084551	0.180	0.684		27172742	27176974		3	1433	-0.037	
H19	NONHSAT017460	0.273	0.152	11	2016405	2018447	Negative	4	1771	-0.139	[18]
	NONHSAT017461	0.720	0.275		2016405	2022940		6	2767	-0.132	
	NONHSAT017462	1.832	0.152		2016405	2019044		5	2281	-0.131	
	NONHSAT017465	1.306	0.189		2016405	2019105		5	2348	-0.131	
	NONHSAT017466	2.880	0.080		2016407	2019072		6	2090	-0.128	

	NONHSAT017467	0.287	0.427		2016439	2019027		3	1796	-0.087	
	NONHSAT017469	0.992	0.303		2016463	2018255		5	1444	-0.21	
	NONHSAT017471	1.801	0.122		2016466	2019045		6	1481	-0.153	
	NONHSAT017472	2.888	ID		2016668	2017801		4	861	-0.059	
	NONHSAT017474	1.006	0.157		2016917	2017697		4	524	-0.068	
HOTAIRM1	NONHSAT119664	7.203	0.907	7	27135712	27139877	Positive	3	1044	-0.159	[19]
	NONHSAT119665	0.250	2.042		27135712	27139877		2	775	-0.138	
	NONHSAT119666	10.453	4.058		27139237	27139884		1	647	-0.003	
	NONHSAT119667	2.488	0.114		27138457	27139758		2	889	-0.101	
Hoxa11as	NONHSAT119711	ID	0.341	7	27227692	27228912	Positive	1	1220	-0.111	[20]
IPW	NONHSAT041066	0.960	0.328	15	25362618	25418143	Positive	6	513	-0.238	[21]
	NONHSAT040981	0.607	0.318		25243100	25265957		8	1677	-0.026	
	NONHSAT040985	0.450	0.168		25245388	25271779		6	6860	-0.183	
	NONHSAT040986	ID	0.340		25245451	25265972		4	470	-0.026	
	NONHSAT040988	0.324	0.433		25223730	25664609		4	17889	-0.083	
	NONHSAT040989	0.332	0.430		25261086	25281638		5	14547	-0.083	
	NONHSAT040990	0.276	IU		25264181	25299063		5	552	-0.31	
	NONHSAT040991	0.456	0.303		25264971	25281638		3	14289	-0.083	
	NONHSAT041000	0.571	0.334		25295778	25367623		33	7844	-0.08	
	NONHSAT041004	0.227	0.263		25299013	25360804		32	4686	-0.056	
	NONHSAT041006	Not detectable	IU		25299859	25300392		2	430	-0.075	
	NONHSAT041023	1.693	0.321		25322012	25328606		5	694	-0.053	
	NONHSAT041028	0.549	0.259		25326300	25359074		17	8842	-0.112	
	NONHSAT041047	0.297	0.502		25340607	25364501		18	2892	-0.202	
	NONHSAT041054	ID	0.685		25354133	25354551		1	418	-0.874	
	NONHSAT041057	0.720	0.339		25354698	25364507		6	1166	-0.218	
	NONHSAT041060	ID	IU		25355629	25362629		4	615	-0.1	

	NONHSAT041061	2.161	0.273		25360161	25364575		4	1224	-0.006	
	NONHSAT041062	1.409	0.273		25360387	25365051		4	1477	-0.204	
	NONHSAT041064	ID	0.409		25362274	25366068		3	2357	-0.127	
	NONHSAT041065	ID	ID		25362556	25420017		7	631	-0.115	
	NONHSAT041140	ID	ID		25580243	25584406		2	1397	-0.245	
Jpx	NONHSAT137572	0.899	3.066	X	73164177	73219338		4	829	-0.146	[22]
	NONHSAT137582	0.248	0.337		73207975	73216331		1	8356	-0.016	
	NONHSAT137583	0.283	0.498		73218247	73224818		2	1146	-0.852	
LINC00568	NONHSAT006301	ID	Not detectable	1	150488232	150490508		2	566	-0.103	[8]
LUCAT1	NONHSAT102744	9.080	1.024	5	90598757	90609474		5	8403	-0.074	[23]
	NONHSAT102748	2.300	0.227		90603570	90610185		4	3860	-0.036	
	NONHSAT102749	3.356	2.040		90603570	90610185		4	5241	-0.085	
	NONHSAT102750	2.952	IU		90606276	90609354		3	1234	-0.08	
linc00467	NONHSAT009289	ID	0.171	1	211556096	211605877		6	758	-0.036	[24]
	NONHSAT009290	0.360	IU		211556144	211570206		5	429	-0.047	
	NONHSAT009291	0.404	0.121		211556157	211572206		5	1236	-0.015	
	NONHSAT009292	0.288	0.677		211556161	211605911		7	1005	-0.038	
	NONHSAT009293	ID	Not detectable		211556178	211565318		4	478	-0.06	
	NONHSAT009294	IU	IU		211564980	211606029		4	651	-0.028	
lincRNA-SFMBT2	NONHSAT011261	ID	IU	10	6821595	6859059	Positive	3	486	-0.184	[25]
lincRNA-VLDLR	NONHSAT129994	ID	IU	9	2422701	2621413	Negative	11	3167	-0.187	[25]
	NONHSAT129993	ID	Not detectable		2422701	2621413		11	3108	-0.187	
	NONHSAT130003	0.710	4.463		2622089	2641395		4	447	-0.07	

lncRNA-HEIH	NONHSAT105843	0.313	1.384	5	180256968	180258971	Positive	2	1497	-0.15	[26]
MEG3	NONHSAT039734	0.289	0.339	14	101245746	101295533	Positive	3	632	-0.064	[27]
	NONHSAT039762	IU	0.682		101295637	101302637		2	2368	-0.153	
	NONHSAT039768	1.801	IU		101298102	101312239		4	4351	-0.13	
	NONHSAT039771	0.360	IU		101299614	101313638		2	4867	-0.127	
	NONHSAT039774	ID	0.546		101301862	101327153		5	721	-0.087	
	NONHSAT039776	ID	Not detectable		101302142	101327096		5	465	-0.237	
MEG9	NONHSAT039999	IU	2.052	14	101536247	101539273	Positive	2	2640	-0.164	[28]
	NONHSAT040000	IU	Not detectable		101536789	101539274		2	742	-0.042	
	NONHSAT040003	IU	Not detectable		101538143	101538895		2	385	-0.113	
NEAT1	NONHSAT022109	IU	0.682	11	65185942	65217564	Positive	2	31379	-0.018	[29]
	NONHSAT022112	0.327	0.318		65190268	65213011		1	22743	-0.205	
	NONHSAT022113	0.363	0.307		65190268	65207625		1	17357	-0.053	
NRON	NONHSAT134764	0.034	0.158	9	129170053	129172783	Negative	1	2730	-0.018	[30]
NTT	NONHSAT115106	0.102	0.231	6	136265388	136282959	Positive	2	17566	-0.032	[31]
Nespas	NONHSAT080575	0.240	IU	20	57392058	57402863	Negative	4	2401	-0.081	[32]
	NONHSAT080583	0.290	0.268		57405764	57414772		2	8886	-0.165	
	NONHSAT080588	0.328	0.379		57416990	57425958		1	8968	-0.059	
ncR-uPAR	NONHSAT102246	ID	0.626	5	76007762	76008686	Positive	1	924	-0.018	[33]

PDZK1IP1-AS1	NONHSAT003057	ID	2.047	1	47644219	47646045	Positive	2	1400	-0.183	[8]
PRINS	NONHSAT011776	IU	ID	10	24536050	24544975	Positive	2	2195	-0.04	[34]
PVT1	NONHSAT129050	0.723	ID	8	128806802	128867565	Positive	3	458	-0.543	[35]
	NONHSAT129049	0.949	0.321		128806799	129113502		18	5132	-0.08	
	NONHSAT129052	1.435	ID		128806805	128951855		3	392	-0.534	
	NONHSAT129054	1.151	0.105		128807999	128903178		2	599	-0.203	
	NONHSAT129055	4.167	ID		128808058	128903041		3	568	-0.106	
	NONHSAT129056	2.397	0.273		128808065	128903206		2	624	-0.157	
	NONHSAT129064	2.881	0.194		128902922	129008896		3	542	-0.071	
	NONHSAT129072	0.241	Not detectable		128996418	129007177		3	211	-0.182	
	NONHSAT129074	ID	0.677		129001462	129108824		4	408	-0.051	
	NONHSAT129085	0.243	0.346		129094932	129113503		3	577	-0.015	
	NONHSAT129088	0.720	IU		129103339	129113488		4	864	-0.084	
Rian	NONHSAT039799	2.081	0.264	14	101361001	101362601	Positive	1	1600	-0.02	[29]
SENCR	NONHSAT025072	3.927	1.368	11	128561566	128565918	Negative	3	1298	-0.151	[36]
SNHG1	NONHSAT021843	4.564	1.500	11	62620543	62621814	Negative	3	625	-0.273	[17]
	NONHSAT021847	0.349	3.829		62621018	62622951		6	532	-0.055	
	NONHSAT021853	IU	1.365		62621839	62622960		3	578	-0.237	
SNHG5	NONHSAT113851	3.596	0.547	6	86375569	86388438	Negative	4	1029	-0.442	[37]
	NONHSAT113863	ID	0.682		86386845	86388451		3	998	-0.425	
ST7OT	NONHSAT122922	ID	ID	7	116712125	116714778	Negative	4	1124	-0.003	[38]

	NONHSAT122927	ID	ID		116785418	116786534		2	592	-0.433	
TUG1	NONHSAT084838	0.241	0.340	22	31368248	31375377	Positive	2	5561	-0.041	[39]

Definition of abbreviations:

CNCI: coding and non-coding indexes.

ID: Infinite time down-regulation (Zero reads from the LPS treatment, but control sample has reads)

IU: Infinite time up-regulation (Zero reads from the control sample, but sample from LPS treatment has reads)

References

[1] J. Yao, B. Zhou, J. Zhang, et al., "A new tumor suppressor LncRNA ADAMTS9-AS2 is regulated by DNMT1 and inhibits migration of glioma cells," *Tumour Biology : The Journal of the International Society for Oncodevelopmental Biology and Medicine*, vol.35, no. 8, pp. 7935-44, 2014.

[2] M. N. Cabili, C. Trapnell, L. Goff, et al., "Integrative annotation of human large intergenic noncoding RNAs reveals global properties and specific subclasses," *Genes and Developement*, vol.25, no. 18, pp. 1915-27, 2011.

[3] W. Deng, J. Wang, J. Zhang, et al., "TET2 regulates LncRNA-ANRIL expression and inhibits the growth of human gastric cancer cells," *IUBMB Life*, vol.68, no. 5, pp. 355-64, 2016.

[4] L. Chen, W. Wang, L. Cao, et al., "Long Non-Coding RNA CCAT1 Acts as a Competing Endogenous RNA to Regulate Cell Growth and Differentiation in Acute Myeloid Leukemia," *Molecules and Cells*, vol.39, no. 4, pp. 330-6, 2016.

[5] S. Memczak, M. Jens, A. Elefsinioti, et al., "Circular RNAs are a large class of animal RNAs with regulatory potency," *Nature*, vol.495, no. 7441, pp. 333-8, 2013.

[6] B. C. Ellis, P. L. Molloy, and L. D. Graham, "CRNDE: A Long Non-Coding RNA Involved in CanceR, Neurobiology, and DEvelopment," *Frontiers in Genetics*, vol.3, no., pp. 270, 2012.

[7] K. Takayama, K. Horie-Inoue, S. Katayama, et al., "Androgen-responsive long noncoding RNA CTBP1-AS promotes prostate cancer," *The EMBO Journal*, vol.32, no. 12, pp. 1665-80, 2013.

[8] U. A. Orom, T. Derrien, M. Beringer, et al., "Long noncoding RNAs with enhancer-like function in human cells," *Cell*, vol.143, no. 1, pp. 46-58, 2010.

[9] I. Ulitsky, A. Shkumatava, C. H. Jan, et al., "Conserved function of lincRNAs in vertebrate embryonic development despite rapid sequence evolution," *Cell*, vol.147, no. 7, pp. 1537-50, 2011.

[10] I. Martianov, A. Ramadass, A. Serra Barros, et al., "Repression of the human dihydrofolate reductase gene by a non-coding interfering transcript," *Nature*, vol.445, no. 7128, pp. 666-70, 2007.

[11] Q. Li, Z. Su, X. Xu, et al., "AS1DHRS4, a head-to-head natural antisense transcript, silences the DHRS4 gene cluster in cis and trans," *Proceedings of the National Academy of Sciences of the United States of America*, vol.109, no. 35, pp. 14110-5, 2012.

[12] J. K. Millar, J. C. Wilson-Annan, S. Anderson, et al., "Disruption of two novel genes by a translocation co-segregating with schizophrenia," *Human Molecular Genetics*, vol.9, no. 9, pp. 1415-23, 2000.

[13] A. Garding, N. Bhattacharya, R. Claus, et al., "Epigenetic upregulation of lncRNAs at 13q14.3 in leukemia is linked to the In Cis downregulation of a gene cluster that targets NF-kB," *PLoS Genetics*, vol.9, no. 4, pp. e1003373, 2013.

[14] M. Lerner, M. Harada, J. Loven, et al., "DLEU2, frequently deleted in malignancy, functions as a critical host gene of the cell cycle inhibitory microRNAs miR-15a and miR-16-1," *Experimental Cell Research*, vol.315, no. 17, pp. 2941-52, 2009.

[15] L. A. Wagner, C. J. Christensen, D. M. Dunn, et al., "EGO, a novel, noncoding RNA gene, regulates eosinophil granule protein transcript expression," *Blood*, vol.109, no. 12, pp. 5191-8, 2007.

[16] M. Mourtada-Maarabouni, A. M. Hasan, F. Farzaneh, et al., "Inhibition of human T-cell proliferation by mammalian target of rapamycin (mTOR) antagonists requires noncoding RNA growth-arrest-specific transcript 5 (GAS5)," *Molecular Pharmacology*, vol.78, no. 1, pp. 19-28, 2010.

[17] T. R. Mercer, I. A. Qureshi, S. Gokhan, et al., "Long noncoding RNAs in neuronal-glial fate specification and oligodendrocyte lineage maturation," *BMC Neuroscience*, vol.11, no., pp. 14, 2010.

[18] A. Gabory, H. Jammes, and L. Dandolo, "The H19 locus: role of an imprinted non-coding RNA in growth and development," *BioEssays : News and Reviews in Molecular, Cellular and Developmental Biology*, vol.32, no. 6, pp. 473-80, 2010.

[19] X. Zhang, Z. Lian, C. Padden, et al., "A myelopoiesis-associated regulatory intergenic noncoding RNA transcript within the human HOXA cluster," *Blood*, vol.113, no. 11, pp. 2526-34, 2009.

[20] H. M. Hsieh-Li, D. P. Witte, M. Weinstein, et al., "Hoxa 11 structure, extensive antisense transcription, and function in male and female fertility," *Development*, vol.121, no. 5, pp. 1373-85, 1995.

[21] J. Rachmilewitz, M. Elkin, L. H. Looijenga, et al., "Characterization of the imprinted IPW gene: allelic expression in normal and tumorigenic human tissues," *Oncogene*, vol.13, no. 8, pp. 1687-92, 1996.

[22] C. Chureau, M. Prissette, A. Bourdet, et al., "Comparative sequence analysis of the X-inactivation center region in mouse, human, and bovine," *Genome Research*, vol.12, no. 6, pp. 894-908, 2002.

[23] P. Thai, S. Statt, C. H. Chen, et al., "Characterization of a novel long noncoding RNA, SCAL1, induced by cigarette smoke and elevated in lung cancer cell lines," *American Journal of Respiratory Cell and Molecular Biology*, vol.49, no. 2, pp. 204-11, 2013.

[24] B. Atmadibrata, P. Y. Liu, N. Sokolowski, et al., "The novel long noncoding RNA linc00467 promotes cell survival but is down-regulated by N-Myc," *PloS One*, vol.9, no. 2, pp. e88112, 2014.

[25] S. Loewer, M. N. Cabili, M. Guttman, et al., "Large intergenic non-coding RNA-RoR modulates reprogramming of human induced pluripotent stem cells," *Nature Genetics*, vol.42, no. 12, pp. 1113-7, 2010.

[26] F. Yang, L. Zhang, X. S. Huo, et al., "Long noncoding RNA high expression in hepatocellular carcinoma facilitates tumor growth through enhancer of zeste homolog 2 in humans," *Hepatology*, vol.54, no. 5, pp. 1679-89, 2011.

[27] X. Zhang, Y. Zhou, K. R. Mehta, et al., "A pituitary-derived MEG3 isoform functions as a growth suppressor in tumor cells," *The Journal of Clinical Endocrinology and Metabolism*, vol.88, no. 11, pp. 5119-26, 2003.

[28] J. P. Hagan, B. L. O'Neill, C. L. Stewart, et al., "At least ten genes define the imprinted Dlk1-Dio3 cluster on mouse chromosome 12qF1," *PloS One*, vol.4, no. 2, pp. e4352, 2009.

[29] M. Guttman, J. Donaghey, B. W. Carey, et al., "lincRNAs act in the circuitry controlling pluripotency and differentiation," *Nature*, vol.477, no. 7364, pp. 295-300, 2011.

[30] A. T. Willingham, A. P. Orth, S. Batalov, et al., "A strategy for probing the function of noncoding RNAs finds a repressor of NFAT," *Science*, vol.309, no. 5740, pp. 1570-3, 2005.

[31] A. Y. Liu, B. S. Torchia, B. R. Migeon, et al., "The human NTT gene: identification of a novel 17-kb noncoding nuclear RNA expressed in activated CD4+ T cells," *Genomics*, vol.39, no. 2, pp. 171-84, 1997.

[32] S. Chillambhi, S. Turan, D. Y. Hwang, et al., "Deletion of the noncoding GNAS antisense transcript causes pseudohypoparathyroidism type Ib and biparental defects of GNAS methylation in cis," *The Journal of Clinical Endocrinology and Metabolism*, vol.95, no. 8, pp. 3993-4002, 2010.

[33] N. R. Madamanchi, Z. Y. Hu, F. Li, et al., "A noncoding RNA regulates human protease-activated receptor-1 gene during embryogenesis," *Biochimica et Biophysica Acta*, vol.1576, no. 3, pp. 237-45, 2002.

[34] E. Sonkoly, Z. Bata-Csorgo, A. Pivarcsi, et al., "Identification and characterization of a novel, psoriasis susceptibility-related noncoding RNA gene, PRINS," *The Journal of Biological Chemistry*, vol.280, no. 25, pp. 24159-67, 2005.

[35] W. J. Cao, H. L. Wu, B. S. He, et al., "Analysis of long non-coding RNA expression profiles in gastric cancer," *World Journal of Gastroenterology*, vol.19, no. 23, pp. 3658-64, 2013.

[36] R. D. Bell, X. Long, M. Lin, et al., "Identification and initial functional characterization of a human vascular cell-enriched long noncoding RNA," *Arteriosclerosis, Thrombosis, and Vascular Biology*, vol.34, no. 6, pp. 1249-59, 2014.

[37] R. Tanaka, H. Satoh, M. Moriyama, et al., "Intronic U50 small-nucleolar-RNA (snoRNA) host gene of no protein-coding potential is mapped at the chromosome breakpoint t(3;6)(q27;q15) of human B-cell lymphoma," *Genes to Cells : Devoted to Molecular & Cellular Mechanisms*, vol.5, no. 4, pp. 277-87, 2000.

[38] J. L. Vincent, D. R. Nelson, and M. D. Williams, "Is worsening multiple organ failure the cause of death in patients with severe sepsis?," *Critical Care Medicine*, vol.39, no. 5, pp. 1050-5, 2011.

[39] A. M. Khalil, M. Guttman, M. Huarte, et al., "Many human large intergenic noncoding RNAs associate with chromatin-modifying complexes and affect gene expression," *Proceedings of the National Academy of Sciences of the United States of America*, vol.106, no. 28, pp. 11667-72, 2009.

Supplementary Table 3: Expression of Protein coding genes (PCGs) overlapping with functionally annotated lncRNAs at 3 and 24 h LPS treatment in human microvascular endothelial cells (HMECs).

Functionally annotated lncRNAs	Overlapping regulatory NONCODE splice variants	Class	PCG	Fold regulation of PCG (3 h)	Fold regulation of PCG (24 h)
ADAMTS9-AS2	NONHSAT090266, NONHSAT090274	Antisense	ADAMTS9	**0.327**	0.950
ANRIL	NONHSAT130413, NONHSAT130414, NONHSAT130416, NONHSAT130417, NONHSAT130422, NONHSAT130423, NONHSAT130425, NONHSAT130433	Antisense	CDKN2B	**0.327**	0.950
CCAT1	NONHSAT129019	Antisense	POU5F1B	ND	ND
CDR1-AS	NONHSAT138820	Antisense	CDR1	0.654	2.036
CYP4A22-AS1	NONHSAT003050, NONHSAT003054	Antisense	CYP4A22	ND	ND
Cyrano	NONHSAT041921, NONHSAT041926	Antisense	OIP5	0.751	1.029
DHFR-upstream-transcripts	NONHSAT102417	Antisense; Exonic	MSH3<br>DHFR	0.618<br>ND	1.050<br>ND

DHRS4-AS1	NONHSAT035952, NONHSAT035953, NONHSAT035955	Antisense	DHRS4L2	1.059	1.134
			DHRS4	0.981	0.898
DISC2	NONHSAT010195	Exonic	DISC1	0.529	1.215
DLEU1	NONHSAT033809, NONHSAT033813, NONHSAT033815, NONHSAT033822, NONHSAT033823, NONHSAT033850	Antisense	DLEU7	ND	ND
DLEU2	NONHSAT033771, NONHSAT033774, NONHSAT033780, NONHSAT033788,	Antisense	KCNRG	**0.268**	1.901
	NONHSAT033796, NONHSAT033798		TRIM13	0.617	1.490
	NONHSAT033800, NONHSAT033805		SPRYD7	0.548	0.839
EGO	NONHSAT087635, NONHSAT087634	Antisense	ITPR1	0.818	1.444
GAS5	NONHSAT007665, NONHSAT007698, NONHSAT007698	Antisense	ZBTB37	0.601	1.370
Hoxa11as	NONHSAT119711	Antisense	hoxa11a	**0.274**	0.839
IPW	NONHSAT041066, NONHSAT040981, NONHSAT040985, NONHSAT040986, NONHSAT040988, NONHSAT040989 NONHSAT040990, NONHSAT040991, NONHSAT041000, NONHSAT041004, NONHSAT041006, NONHSAT041023, NONHSAT041028, NONHSAT041047, NONHSAT041054, NONHSAT041057, NONHSAT041060, NONHSAT041061, NONHSAT041062, NONHSAT041064, NONHSAT041065, NONHSAT041140	Antisense	UBE3A	ND	ND

lincRNA-VLDLR	NONHSAT129994, NONHSAT129993, NONHSAT130003	Antisense	VLDLR	0.461	0.557
NRON	NONHSAT134764	Antisense	MVB12B	0.352	1.808
NTT	NONHSAT115106	Exonic	PDE7B	**0.068**	ND
Nespas	NONHSAT080575, NONHSAT080583, NONHSAT080588	Antisense	GNAS	0.865	1.465
PRINS	NONHSAT011776	Sense No Exonic	KIAA1217	0.654	1.742
SENCR	NONHSAT025072	Antisense	FLI1	1.229	1.368
ST7OT	NONHSAT122922, NONHSAT122927	Antisense	ST7	0.695	1.014

Definition of abbreviation:

PCG: Protein coding gene

Supplementary Table 4: Significant canonical pathways and associated differentially regulated protein coding genes (PCGs) at 3 h in human microvascular endothelial cells (HMECs) after LPS treatment.

Ingenuity Canonical Pathways	PCGs involved
Granulocyte Adhesion and Diapedesis	CXCL8,IL1A,VCAM1,ICAM1,MMP3,CCL20,SDC4,CCL5,CCL11,HRH1,CXCL3,CCL2,IL1B, CXCL1,TNFRSF11B
Role of Macrophages, Fibroblasts and Endothelial Cells in Rheumatoid Arthritis	CXCL8,VCAM1,IL1A,ICAM1,MMP3,FGF2,IL32,VEGFC,IL6,CCL5,TLR2,JUN,NFKBIA, CCL2, IL1B,FZD5,TNFSF13B,TNFRSF11B
Agranulocyte Adhesion and Diapedesis	CXCL8,IL1A,VCAM1,ICAM1,MMP3,CCL20,SDC4,CCL5,CCL11,HRH1,CXCL3,CCL2,IL1B, CXCL1
Atherosclerosis Signaling	CXCL8,IL1A,VCAM1,ICAM1,CCL2,MMP3,CD40,IL1B,IL6,CCL11,F3
Glucocorticoid Receptor Signaling	CXCL8,VCAM1,ICAM1,SMAD3,CCL5,IL6,CCL11,CXCL3,NFKBIA,JUN,CCL2,IL1B,PTGS, PLAU,SERPINE1
Role of IL-17A in Arthritis	CXCL8,CXCL3,NFKBIA,CCL2,CCL20,CXCL1,CCL5,PTGS2
Activation of IRF by Cytosolic Pattern Recognition Receptors	IFIH1,JUN,NFKBIA,CD40,IL6,ISG15
Role of Hypercytokinemia/hyperchemokinemia in the Pathogenesis of Influenza	CXCL8,IL1A,CCL2,IL1B,CCL5,IL6
IL-17 Signaling	CXCL8,JUN,CCL2,MMP3,CXCL1,PTGS2,IL6,CCL11
Role of IL-17A in Psoriasis	CXCL8,CXCL3,CCL20,CXCL1

Death Receptor Signaling	NFKBIA,TNFRSF10B,TNFSF10,TNFSF15,CFLAR,BIRC3,BIRC2
HMGB1 Signaling	CXCL8,IL1A,VCAM1,ICAM1,JUN,CCL2,IL1B,IL6,SERPINE1,TNFRSF11B
Hepatic Fibrosis / Hepatic Stellate Cell Activation	CXCL8,IL1A,VCAM1,ICAM1,FGF2,SMAD3,VEGFC,IL6,CCL5,CXCL3,CD40,CCL2,IL1B, SERPINE1,TNFRSF11B
Differential Regulation of Cytokine Production in Macrophages and T Helper Cells by IL-17A and IL-17F	CCL2,IL1B,CXCL1,CCL5,IL6
TNFR2 Signaling	JUN,NFKBIA,BIRC3,BIRC2
TREM1 Signaling	TLR2,CXCL8,CXCL3,ICAM1,CCL2,CD40,IL1B,IL6
Differential Regulation of Cytokine Production in Intestinal Epithelial Cells by IL-17A and IL-17F	IL1A,CCL2,IL1B,CXCL1,CCL5
Communication between Innate and Adaptive Immune Cells	TLR2,CXCL8,IL1A,CD40,IL1B,CCL5,IL6,TNFSF13B
Acute Phase Response Signaling	IL1A,SOD2,JUN,C3,NFKBIA,IL1B,IL6,SERPINE1,TNFRSF11B
Role of Cytokines in Mediating Communication between Immune Cells	CXCL8,IL1A,IL32,IL1B,IL6
Role of IL-17F in Allergic Inflammatory Airway Diseases	CXCL8,CCL2,IL1B,CXCL1,IL6
TNFR1 Signaling	JUN,NFKBIA,BIRC3,BIRC2
Dendritic Cell Maturation	TLR2,IL1A,ICAM1,NFKBIA,CD40,IL32,IL1B,IL6,TNFRSF11B
IL-17A Signaling in Gastric Cells	CXCL8,JUN,CCL20,CXCL1,CCL5
IL-17A Signaling in Airway Cells	CXCL3,NFKBIA,CCL20,CXCL1,IL6,CCL11
Inflammasome pathway	CXCL8,IL1B,P2RX7

Role of Pattern Recognition Receptors in Recognition of Bacteria and Viruses	TLR2,IFIH1,CXCL8,IL1A,C3,IL1B,CCL5,IL6
IL-6 Signaling	CXCL8,IL1A,JUN,NFKBIA,IL1B,IL6,TNFRSF11B
TWEAK Signaling	NFKBIA,BIRC3,BIRC2
Systemic Lupus Erythematosus Signaling	IL1A,JUN,CD40,IL1B,IL6,TNFSF13B
CD40 Signaling	ICAM1,JUN,NFKBIA,CD40,PTGS2
Hepatic Cholestasis	CXCL8,IL1A,JUN,NFKBIA,IL1B,IL6,TNFRSF11B
LXR/RXR Activation	IL1A,C3,CCL2,IL1B,PTGS2,IL6,TNFRSF11B
Retinoic acid Mediated Apoptosis Signaling	TNFRSF10B,TNFSF10,CFLAR
Lymphotoxin β Receptor Signaling	VCAM1,NFKBIA,CXCL1,BIRC2
IL-17A Signaling in Fibroblasts	JUN,NFKBIA,CCL2,IL6
PPAR Signaling	IL1A,JUN,NFKBIA,IL1B,PTGS2,TNFRSF11B
Eicosanoid Signaling	PTGFR,PTGIR,PTGS2,PTGDS,PTGER4
Hematopoiesis from Pluripotent Stem Cells	CXCL8,IL1A,IL6
Altered T Cell and B Cell Signaling in Rheumatoid Arthritis	TLR2,IL1A,CD40,IL1B,IL6,TNFSF13B
Coagulation System	PLAU,SERPINE1,F3
B Cell Activating Factor Signaling	JUN,NFKBIA,TNFSF13B
Role of Osteoblasts, Osteoclasts and Chondrocytes in Rheumatoid Arthritis	IL1A,JUN,NFKBIA,MMP3,IL1B,FZD5,IL6,BIRC3,TNFRSF11B,BIRC2
Induction of Apoptosis by HIV1	NFKBIA,BIRC3,TNFRSF11B,BIRC2

Cholecystokinin/Gastrin-mediated Signaling	IL1A,JUN,IL1B,PTGS2
PI3K Signaling in B Lymphocytes	JUN,C3,NFKBIA,CD40
Toll-like Receptor Signaling	TLR2,IL1A,JUN,NFKBIA,IL1B

Supplementary Table 5: Significant canonical pathways and associated differentially regulated protein coding genes (PCGs) at 24 h in human microvascular endothelial cells (HMECs) after LPS treatment.

Ingenuity Canonical Pathways	Genes involved
Role of IL-17F in Allergic Inflammatory Airway Diseases	CXCL10,CXCL8,IGF1,CCL2,IL1B,CXCL1,CXCL5,IL6,CSF2
Role of IL-17A in Arthritis	CXCL8,CXCL3,NFKBIA,CCL2,CCL20,CXCL1,CXCL5,CCL5,PTGS2,MMP1
Agranulocyte Adhesion and Diapedesis	AOC3,CXCL8,SELE,IL1A,VCAM1,ICAM1,CXCR4,CCL20,CXCL5,CCL5,CXCL10,CXCL3,CCL2,IL1B, CXCL1, MMP1
Granulocyte Adhesion and Diapedesis	CXCL8,SELE,IL1A,VCAM1,ICAM1,CXCR4,CCL20,CXCL5,CCL5,CXCL10,CXCL3,CCL2,IL1B,CXCL1, MMP1,TNFRSF11B
Role of IL-17A in Psoriasis	CXCL8,CXCL3,CCL20,CXCL1,CXCL5
Role of Hypercytokinemia/hyperchemokinemia in the Pathogenesis of Influenza	CXCL10,CXCL8,IL1A,CCL2,IL1B,CCL5,IL6
Role of Macrophages, Fibroblasts and Endothelial Cells in Rheumatoid Arthritis	CXCL8,VCAM1,SELE,IL1A,ICAM1,IL32,VEGFC,IL6,CCL5,NFKBIA,CCL2,IL1B,CSF2,TNFSF13B, LRP1, MMP1,TNFRSF11B,CAMK2B
Communication between Innate and Adaptive Immune Cells	CXCL10,HLA-G,CXCL8,IL1A,HLA-B,IL1B,CCL5,IL6,CSF2,TNFSF13B

Differential Regulation of Cytokine Production in Macrophages and T Helper Cells by IL-17A and IL-17F	CCL2,IL1B,CXCL1,CCL5,IL6,CSF2
HMGB1 Signaling	CXCL8,SELE,IL1A,VCAM1,ICAM1,CCL2,IL1B,IL6,SERPINE1,CSF2,TNFRSF11B
Differential Regulation of Cytokine Production in Intestinal Epithelial Cells by IL-17A and IL-17F	IL1A,CCL2,IL1B,CXCL1,CCL5,CSF2
Role of Cytokines in Mediating Communication between Immune Cells	CXCL8,IL1A,IL32,IL1B,IL6,CSF2
Glucocorticoid Receptor Signaling	CXCL8,SELE,VCAM1,ICAM1,CCL5,IL6,CXCL3,NFKBIA,CCL2,IL1B,PTGS2,CSF2,SERPINE1,MMP1
Atherosclerosis Signaling	CXCL8,SELE,IL1A,VCAM1,ICAM1,CCL2,CXCR4,IL1B,IL6,MMP1
Retinol Biosynthesis	AKR1C3,LIPG,RBP1
Hepatic Fibrosis / Hepatic Stellate Cell Activation	CXCL3,CXCL8,VCAM1,IL1A,ICAM1,CCL2,IGF1,IL1B,VEGFC,CCL5,IL6,SERPINE1,MMP1,TNFRSF11 B
Acute Phase Response Signaling	IL1A,SOD2,C3,NFKBIA,IL1B,IL6,SERPINE1,RBP1,TNFRSF11B
IL-17 Signaling	CXCL10,CXCL8,CCL2,CXCL1,CXCL5,PTGS2,IL6
Graft-versus-Host Disease Signaling	HLA-G,IL1A,HLA-B,IL1B,IL6
IL-17A Signaling in Fibroblasts	NFKBIA,CCL2,CXCL5,IL6,MMP1
Hematopoiesis from Pluripotent Stem Cells	CXCL8,IL1A,IL6,CSF2
Dendritic Cell Maturation	IL1A,ICAM1,NFKBIA,IL32,HLA-B,IL1B,IL6,CSF2,TNFRSF11B
TREM1 Signaling	CXCL8,CXCL3,ICAM1,CCL2,IL1B,IL6,CSF2
IL-17A Signaling in Gastric Cells	CXCL10,CXCL8,CCL20,CXCL1,CCL5

IL-17A Signaling in Airway Cells	CXCL3,NFKBIA,CCL20,CXCL1,CXCL5,IL6
The Visual Cycle	AKR1C3,RBP1
Role of Pattern Recognition Receptors in Recognition of Bacteria and Viruses	IFIH1,CXCL8,IL1A,C3,IL1B,CCL5,IL6,CSF2
Altered T Cell and B Cell Signaling in Rheumatoid Arthritis	IL1A,SPP1,HLA-B,IL1B,IL6,CSF2,TNFSF13B
Role of Osteoblasts, Osteoclasts and Chondrocytes in Rheumatoid Arthritis	IL1A,SPP1,NFKBIA,IGF1,IL1B,IL6,CSF2,BIRC3,LRP1,MMP1,TNFRSF11B
Systemic Lupus Erythematosus Signaling	HLA-G,IL1A,HLA-B,IL1B,IL6,TNFSF13B
Activation of IRF by Cytosolic Pattern Recognition Receptors	IFIH1,NFKBIA,IL6,ISG15
Role of MAPK Signaling in the Pathogenesis of Influenza	CXCL10,CCL2,CCL5,PTGS2
Hepatic Cholestasis	CXCL8,IL1A,NFKBIA,IL1B,IL6,CSF2,TNFRSF11B
LXR/RXR Activation	IL1A,C3,CCL2,IL1B,PTGS2,IL6,TNFRSF11B
Retinoate Biosynthesis I	AKR1C3,RBP1
Bile Acid Biosynthesis, Neutral Pathway	AKR1C1/AKR1C2,AKR1C3
Methylglyoxal Degradation III	AKR1C1/AKR1C2,AKR1C3
Airway Pathology in Chronic Obstructive Pulmonary Disease	CXCL8,CXCL3,MMP1
IL-6 Signaling	CXCL8,IL1A,NFKBIA,IL1B,IL6,TNFRSF11B

Crosstalk between Dendritic Cells and Natural Killer Cells	HLA-G,IL3RA,HLA-B,IL6,CSF2,CAMK2B
Triacylglycerol Degradation	NDST3,LIPG
Induction of Apoptosis by HIV1	NFKBIA,CXCR4,BIRC3,TNFRSF11B
Cholecystokinin/Gastrin-mediated Signaling	IL1A,IL1B,EPHA4,PTGS2
OX40 Signaling Pathway	HLA-G,NFKBIA,HLA-B
Chemokine Signaling	CCL2,CXCR4,CCL5,CAMK2B
Role of Tissue Factor in Cancer	CXCL8,VEGFC,IL1B,CXCL1,CSF2,MMP1
Antigen Presentation Pathway	HLA-G,HLA-B
PPAR Signaling	IL1A,NFKBIA,IL1B,PTGS2,TNFRSF11B
